# Checking for model failure and for prior-data conflict with the constrained multinomial model

**DOI:** 10.1007/s00184-021-00811-8

**Published:** 2021-03-03

**Authors:** Berthold-Georg Englert, Michael Evans, Gun Ho Jang, Hui Khoon Ng, David Nott, Yi-Lin Seah

**Affiliations:** 1 Centre for Quantum Technologies, Department of Physics, National University of Singapore and MajuLab, Singapore, Singapore; 2 Department of Statistical Sciences, University of Toronto, Toronto, ON Canada; 3 Ontario Institute for Cancer Research, Toronto, ON Canada; 4 Yale-NUS College, Centre for Quantum Technologies, MajuLab, Singapore, Singapore; 5 Department of Statistics and Applied Probability, National Unversity of Singapore, Singapore, Singapore; 6 Centre for Quantum Technologies, Singapore, Singapore

**Keywords:** Model checking, Checking for prior-data conflict, Quantum state estimation, Hardy–Weinberg equilibrium, Ordered probabilities, Elicitation

## Abstract

Multinomial models can be difficult to use when constraints are placed on the probabilities. An exact model checking procedure for such models is developed based on a uniform prior on the full multinomial model. For inference, a nonuniform prior can be used and a consistency theorem is proved concerning a check for prior-data conflict with the chosen prior. Applications are presented and a new elicitation methodology is developed for multinomial models with ordered probabilities.

## Introduction

Suppose we have a sample of *n* from a multinomial$(1,\theta _{1},\ldots ,\theta _{k+1})$ distribution and let $T=(T_{1},\ldots ,T_{k+1})$ denote the cell counts. There are a number of applications where constraints are placed on $\theta =(\theta _{1},\ldots ,\theta _{k+1})$ such that it lies in some proper subset ${\varTheta }$ of the full *k*-dimensional simplex ${\varTheta }_{k}$, see Sect. 2. This is the constrained multinomial model. Also, there are applications where there is additional information concerning $\theta$ that takes the form of a prior probability measure ${\varPi }$ on ${\varTheta }$. Our goal here is to check the model, namely, see if the data indicate that the true $\theta$ lies outside ${\varTheta }$, and if the model is found acceptable, then check the prior, namely, see if the data indicate that the true $\theta$ lies in a region of relatively low prior probability in ${\varTheta }$.

The primary way to determine whether or not an ingredient to a statistical analysis is appropriate is to compare it somehow with the data. For example, if the observed data is surprising for every distribution in the model, then it is reasonable to question the appropriateness of the model. By surprising it is meant that the data falls in a region such that each distribution in the model gives a relatively low probability for that data’s occurrence. Once a model is accepted, a chosen prior is questionable if there is an indication that the true value lies in the tails of the prior. For example, if prior measure ${\varPi }$ with density $\pi$ is used for a model parameter $\theta$ with true value $\theta ^{\text {true}}$, then a prior is placing its mass in the wrong part of the parameter space ${\varTheta }$ if ${\varPi }(\pi (\theta )\le \pi (\theta ^{\text {true}}))$ is small. While we cannot know this quantity, an assessment of this can be made based on the data, as discussed in Sect. 4, which is in itself an assessment of whether or not the observed data is incompatible with its prior distribution, namely, whether or not a prior-data conflict exists. As discussed in Al Labadi and Evans (2017b), inferences can be quite sensitive to the choice of the prior when prior-data conflict exists.

While a simultaneous check on the model and prior can be considered, as in Box (1980), separate checks are developed here so that we can identify whether an identified problem lies with the model or with the prior. Also, the check on the prior is performed only if the model has not failed its check, as the prior is implicitly dependent on the model. As discussed in Evans and Moshonov (2006, 2007), the check on the prior can sometimes be further decomposed so that individual components of the prior can be checked. So there is an overall logic for checking the ingredients to a Bayesian statistical analysis.

A notable aspect of the model checking approach taken here is that it is Bayesian but does not involve the prior ${\varPi }$. Rather we consider first the multinomial model with no constraints and place a uniform prior ${\varPi }_{k}$ on ${\varTheta }_{k}$. Then, based on a distance measure $d(\theta ,\theta ^{\prime })$ defined on ${\varTheta }_{k}^{2}$, the prior distribution of the distance $d_{{\varTheta }}(\theta )=\inf _{\theta ^{\prime }\in {\varTheta }}d(\theta ,\theta ^{\prime })$ is compared with its posterior distribution. In essence, if the posterior has concentrated less about ${\varTheta }$ than the prior, then there is evidence against the constrained model and if the posterior has concentrated more about ${\varTheta }$ than the prior, then there is evidence in favor of the constrained model. There are several advantages possessed by this approach. First, up to simulation errors, the assessment is exact and does not depend upon asymptotics. Second, the approach works even when ${\varTheta }$ is a complicated lower dimensional subset of ${\varTheta }_{k}$ and it is straightforward to implement provided $d_{{\varTheta }}(\theta )$ is relatively easy to compute. Of course, we have the freedom to choose *d* to address the latter concern. Finally, it is possible to obtain evidence in favor of the constrained multinomial and not just evidence against as is common with most goodness of fit tests. This makes sense as, provided there is *i*.*i*.*d*. sampling, then some multinomial distribution is correct. A check for *i*.*i*.*d*. sampling can be made by generalizations of the runs test, but we always assume that such sampling is correct here. Model checking is discussed in Sect. 3.

The check for the prior is based on the approach taken in Evans and Moshonov (2006) and is discussed in Sect. 4. The consistency of this check was established in Evans and Jang (2011a) under fairly general conditions. These conditions do not hold for the constrained multinomial, however, and so a consistency theorem is proved for this context. A new result is established for putting a prior on ordered probabilities which arise in many important applications. Computational issues are addressed in the examples. A high level of numerical accuracy is generally not required, however, as knowing relevant probabilities to one or two decimal places is sufficient to detect whether or not a problem exists.

Box (1980) is a key paper in Bayesian model checking. One notable aspect of this paper is that the check is based on the prior predictive distribution of the data, namely, the distribution of the data as induced by the sampling model and the prior distribution. There are also approaches to model checking that are based upon the posterior predictive distribution of the data such as Guttman (1967), Rubin (1984) and Gelman et al. (1996). A different approach is taken in Bayarri and Berger (2000) and Castellanos and Bayarri (2007) where the check is based on the conditional distribution of the data given a statistic that is asymptotically minimally sufficient. These checks are similar to frequentist model checks based on the conditional distribution of the data given a minimal sufficient, which is independent of the model parameter. As noted in Robins et al. (2000), there is a problem with posterior predictive checks when based on p-values as these fail to have to have asymptotically uniform distributions when the model is correct and so can be misleading. The Bayarri and Berger (2000) checks do not suffer from this defect. There have been attempts to recalibrate posterior p-values as in Hjort et al. (2006) and van Kollenburg et al. (2017). There is no reason to suppose, however, that model checks need to be a posteriori or that p-values need to be used as part of this. Posterior distributions are used in inferences about parameters as necessitated by the principle of conditional probability which says that prior beliefs must be updated via conditioning on the observed data. But this says nothing about how checks on the sampling model or the prior should be conducted. Also, there are many issues that lead to doubts about whether or not p-values are valid expressions of evidence. In the case of the constrained multinomial it is natural to put a uniform prior on the full multinomial model and then, as discussed in Sect. 3, assess the submodel based upon a valid measure of evidence.

Several different approaches have been developed for checking the prior. Bousquet (2008) bases such a check on a comparison with an improper reference prior. Presanis et al. (2013) develop diagnostics for such checks. Nott et al. (2020) develops an extension to the approach of Evans and Moshonov (2006) that provides a check with an invariance property necessary for contexts where the data is continuous. The data for the constrained multinomial is discrete so this extension is not required here. Tail probabilities are used for checking the prior, but these are used simply as a measure of surprise and not as a measure of evidence as there is no sense in which a prior is true or false, only that it may be inappropriate.

## Examples

The following examples of constrained multinomial models are considered. The notation *T* in the examples refers to the vector of counts and subscripts on *T* reference the count of a particular cell. Also, the notation *T*(*x*) references the vector of observed counts based on a sample *x*. It is to be noted that *T* is a minimal sufficient statistic for the full multinomial model and so the full data *x* is not necessary for the approach taken here.

### Example 1

Quantum state estimation.

A qubit is any binary quantum system. For example, the spin of the electron, which can be up or down, or the polarization of a photon, which can be polarized vertically or horizontally. Quantum theory allows for these to be held simultaneously, called superposition, and this is represented by a certain operator called the state, in turn represented by a Hermitian unit-trace $2\times 2$ matrix. To estimate the state of such a quantum system, in an experiment, a physical apparatus with $k+1$ detectors is employed that results in one and only one of the detectors recording a click and the *i*-th detector records a click with probability $\theta _{i}$. So a single measurement is a value from a multinomial$(1,\theta _{1},\ldots ,\theta _{k+1})$ distribution. In an experiment, *n* identical copies of the quantum system are used and *i*.*i*.*d*. measurements are obtained. Therefore, with the observed count from the *i*-th detector denoted as $T_{i}(x)$, then $T(x)=(T_{1}(x),\ldots ,T_{k+1}(x))\sim$ multinomial$(n,\theta _{1},\ldots ,\theta _{k+1})$.

Depending on how the measurements are taken, the probabilities must satisfy certain constraints. For example, when $k+1=3$, then the *symmetric trine* model imposes the constraint $\theta _{1}^{2}+\theta _{2}^{2}+\theta _{3}^{2}\le 1/2$, with more involved constraints required for the asymmetric case as discussed in Example 4. When $k+1=4$, then the *cross-hairs* model imposes the constraints $\theta _{1}+\theta _{2}=1/2,\theta _{3}+\theta _{4}=1/2$ and $\theta _{1}^{2}+\theta _{2}^{2}+\theta _{3}^{2}+\theta _{4}^{2} \le 3/8$, while the *tetrahedron* model corresponds to the constraint $\theta _{1}^{2}+\theta _{2}^{2}+\theta _{3}^{2}+\theta _{4}^{2}\le 1/3$ only. When $k+1=6$, then the *Pauli* model imposes the constraints $\theta _{1}+\theta _{2}=1/3,\theta _{3}+\theta _{4}=1/3,\theta _{5}+\theta _{6}=1/3$ and $\theta _{1}^{2}+\theta _{2}^{2}+\theta _{3}^{2}+\theta _{4} ^{2}+\theta _{5}^{2}+\theta _{6}^{2}\le 2/9$. More on how these models arise can be found in Shang et al. (2013). These applications produce a rich variety of constrained multinomial models.

### Example 2

Hardy-Weinberg equilibrium (HWE).

Consider a trait in a population governed by a gene with *l* alleles $A_{1},\ldots ,A_{l}$ with population proportions $\omega _{1},\ldots , \omega _{l}$. There are then $l(l+1)/2$ possible genotypes $A_{i}A_{j}$. Let $\theta _{ij}$ denote the proportion of individuals in the population of genotype $A_{i}A_{j}$ for $$1\le i\le j\le l,\ $$and note that $A_{i}A_{j}$ and $A_{j}A_{i}$ are indistinguishable. Then, under the assumptions of the Hardy-Weinberg equilibrium,

1 $$\begin{aligned} \theta _{ii}=\omega _{i}^{2}\quad \text { and }\quad \theta _{ij}=2\omega _{i}\omega _{j}\quad \text { for }\quad 1\le i<j\le l. \end{aligned}$$

So it is necessary to check if there is evidence for or against (1) holding and this will be based on the observed counts in a sample of *n* where $T_{ij}(x)$ denotes the number of observations having genotype $A_{i}A_{j}$.

### Example 3

Multinomial with ordered probabilities.

Sometimes it is reasonable to suppose that the $\theta _{i}$ satisfy an ordering such as

2 $$\begin{aligned} \theta _{1}\ge \theta _{2}\ge \cdots \ge \theta _{k+1}. \end{aligned}$$

Such a model arises in contexts where systems exhibit aging as in, for example, Briegel et al. (1994). In certain ROC analyses for a medical context, see Zhou et al. (2011), there is a diagnostic variable for a disease which leads to a classification of patients with known disease status into $k+1$ ordered categories. It may be natural to believe that the probability of being in the *i*-th category increases with *i* for diseased patients and decreases for nondiseased patients. In dose-response models, as discussed in Chuang-Stein and Agresti (1997), consider *I* different locations (or treatments) and *J* levels of exposure to a toxic substance from from low to high with $\theta _{ij}$ the probability of a reaction for an individual at location *i* with exposure level *j*. A monotone increasing effect due to increasing exposure leads to $\theta _{i1}\le \cdots \le \theta _{iJ}$ for $i=1,\ldots ,I$. For a constrained multinomial satisfying (2), $T_{i}(x)$ refers to the count for the *i*-th cell.

The following result is useful with this model.

### Lemma 1

Any $\theta \in {\varTheta }_{k}$ satisfying (2) is given by, for some $\omega \in {\varTheta }_{k}$,

3 $$\begin{aligned} (\theta _{1},\ldots ,\theta _{k+1})^{t}=A_{k}(\omega _{1},\ldots ,\omega _{k+1})^{t} \end{aligned}$$

and any $\omega \in {\varTheta }_{k}$ produces a $\theta \in {\varTheta }_{k}$ satisfying (2) via (3), with

$$\begin{aligned} A_{k}=\left( \begin{array}[c]{ccccc} 1 &{} 1/2 &{} 1/3 &{} \ldots &{} 1/(k+1)\\ 0 &{} 1/2 &{} 1/3 &{} \ldots &{} 1/(k+1)\\ 0 &{} 0 &{} 1/3 &{} \ldots &{} 1/(k+1)\\ \vdots &{} \vdots &{} \vdots &{} \vdots &{} \vdots \\ 0 &{} 0 &{} 0 &{} \ldots &{} 1/(k+1) \end{array} \right) . \end{aligned}$$

### Proof

Suppose $(\theta _{1},\ldots ,\theta _{k+1})^{t} =A_{k}(\omega _{1},\ldots ,\omega _{k+1})^{t}$ for $\omega \in {\varTheta }_{k}$. Then $\theta _{i}=\sum _{j=i}^{k+1}\omega _{j}/j$ and it is clear that $0\le \theta _{i}\le 1$ and $\theta _{1}+\cdots +\theta _{k+1}=\omega _{1}+\cdots +\omega _{k+1}=1$ so $\theta \in {\varTheta }_{k}$. It is also immediate that $\theta$ satisfies (2).

If $\theta \in {\varTheta }_{k}$ satisfies (2), put $(\omega _{1} ,\ldots ,\omega _{k+1})^{\prime }=A_{k}^{-1}(\theta _{1},\ldots ,\theta _{k+1})^{t}$ and note

$$\begin{aligned} A_{k}^{-1}=\left( \begin{array} [c]{ccccc} 1 &{} -1 &{} 0 &{} \ldots &{} 0\\ 0 &{} 2 &{} -2 &{} \ldots &{} 0\\ 0 &{} 0 &{} 3 &{} \ldots &{} 0\\ \vdots &{} \vdots &{} \vdots &{} \vdots &{} \vdots \\ 0 &{} 0 &{} 0 &{} \ldots &{} k+1 \end{array} \right) . \end{aligned}$$

Therefore, $\omega _{i}=i(\theta _{i}-\theta _{i+1})$ for $i=1,\ldots ,k$ and $\omega _{k+1}=(k+1)\theta _{k+1}$. Since $\theta _{i}\ge \theta _{i+1}$, then $\omega _{i}\ge 0$ and if $\omega _{i}>1$ then $\theta _{i}>\theta _{i+1}+1/i>1/i$ which by (2) implies $\theta _{1}+\cdots +\theta _{i}>1$ which is false. So $\omega _{i}\in [0,1]$ for $i=1,\ldots ,k$ and similarly $\omega _{k+1}\in [0,1]$. Finally, $\omega _{1}+\cdots +\omega _{k+1} =\sum _{j=1}^{k}i(\theta _{i}-\theta _{i+1})+(k+1)\theta _{k+1}=\theta _{1} +\cdots +\theta _{k+1}=1$. $\square$

A particular model satisfying (2) is given by the Zipf-Mandelbrot distribution where $\theta _{i}=(\alpha +i)^{-\beta }/C_{k}\left( \alpha ,\beta \right)$ for parameters $\alpha >-1,\beta \ge 0$, for $i=1,\ldots ,k+1$ and $C_{k}\left( \alpha ,\beta \right) =\sum _{i=1}^{k+1}(\alpha +i)^{-\beta }$ and denoted here by ${\text {ZM}}_{k}\left( \alpha ,\beta \right)$. When $\beta =0$ this is the uniform distribution and for fixed $\beta$ this converges to the uniform as $\alpha \rightarrow \infty$. For fixed $\alpha$ the distribution becomes degenerate on the first cell as $\beta \rightarrow \infty$. For large *k* with $\alpha =0$ and $\beta >1$, the zeta$(\beta )$ distribution, with $\theta _{i}\propto i^{-\beta }$ for $i=1,2,\ldots$, serves as an approximation. As discussed in Izsák (2006), there are a variety of applications of this distribution as in word frequency distributions in texts.

## Checking the model

In the context of the constrained multinomial model it is possible to use more formal inference methods than are typically used for model checking. The following subsection describes these inferences in some generality and subsequently these are applied to the problem of interest.

### Relative belief inferences

The approach to inference as discussed in Evans (2015) will be employed as this is based upon a direct measure of evidence obtained via the principal of evidence as described, for example, in Achinstein (2000). For example, suppose we have a probability model $(S,{\mathcal {A}},P)$ where $S=\{s_{1},\ldots ,s_{N}\}$ is a finite set, that interest is in whether not the event $A\in {\mathcal {A}}$ has occurred and the event *C*, with $P(C)>0$, has been observed to be true. The principle of evidence then says that there is evidence in favor of *A* being true if $P(A\,|\,C)>P(A)$, there is evidence against *A* being true if $P(A\,|\,C)<P(A)$ and no evidence either way if $P(A\,|\,C)=P(A)$ as then *A* and *C* are independent. So there can be evidence in favor of *A* being true if the belief that *A* is true has increased after being told the information that *C* is true and evidence against *A* being true if the belief that *A* is true has decreased after being told that *C* is true. If *A* and *C* correspond to specifying the value of functions *U* and *V*, say $A=\{s:U(s)=u_{0}\}$ and $C=\{s:V(s)=v_{0}\}$ then, letting $p_{U}$ and $p_{U}(\cdot \,|\,V=v_{0})$ denote the marginal and conditional probability functions of *U*, there is evidence in favor of $u_{0}$ being true when $p_{U}(u_{0}\,|\,V=v_{0})>p_{U}(u_{0})$ or equivalently whenever the *relative belief ratio* $RB_{U} (u_{0}\,|\,V=v_{0})=p_{U}(u_{0}\,|\,V=v_{0})/p_{U}(u_{0})>1$. Note that the cutoff value of 1, for determining whether or not there is evidence in favor or against $u_{0}$ via the relative belief ratio, is not arbitrary, as it is dictated by the principle of evidence.

It would seem that the bigger $RB_{U}(u_{0}\,|\,V=v_{0})$ is than 1, the more evidence there is in favor of $u_{0}$, but this needs to be calibrated. In Evans (2015) this is done via quoting the *strength of the evidence* as

4 $$\begin{aligned} P_{U}(RB_{U}(u\,|\,V=v_{0})\le RB_{U}(u_{0}\,|\,V=v_{0})\,|\,V=v_{0}). \end{aligned}$$

For when $RB_{U}(u_{0}\,|\,V=v_{0})>1$ and (4) is large, there is a small probability that an alternative value of *U* has more evidence in its favor than $u_{0}$ so it is reasonable to regard the value $RB_{U} (u_{0}\,|\,V=v_{0})$ as indicating strong evidence in favor of $u_{0}$ and when (4) is small, there is only weak evidence in favor of $u_{0}$. Similarly, if $RB_{U}(u_{0}\,|\,V=v_{0})<1$ and (4) is small, then there is strong evidence against $u_{0}$, since there is a large probability that an alternative has either more evidence in its favor, or at least less evidence against it, and when (4) is large, there is only weak evidence against $u_{0}$. Note that the relative belief ratio is used here only to order the possible values of *U* with respect to the evidence. Also, alternative measures of the strength of the evidence can be considered. For example, the conditional probability $P(\{u_{0}\}\,|\,V=v_{0})$ is certainly relevant, particularly when the number of possible values of *U* is quite small but in general contexts, (4) works quite well.

For an estimation problem, namely, where it is necessary to choose a value of *U* from the set of possibilities $\{U(s):s\in S\}$ after observing $V=v_{0}$, the natural estimate is to use the *relative belief estimate* $u(v_{0})=\arg \max RB_{U}(u\,|\,V=v_{0})$ as this maximizes the evidence in favor. The plausible region $Pl(v_{0})=\{u:RB_{U}(u\,|\,V=v_{0})>1\}$, namely, the set of values of *u* for which evidence in favor has been obtained, can be used to assess the accuracy of $u(v_{0})$, via the size of $Pl(v_{0})$ as well as its conditional probability $P_{U}(Pl(v_{0})\,|\,V=v_{0})$.

While the simple finite context just described gives the basic ideas underlying the approach to inference adopted here, this can be generalized to infinite discrete models in an obvious way and also to continuous models via taking limits of sets shrinking to points as discussed in Evans (2015). In Bayesian contexts with a proper prior, then there is a joint probability model for the model parameter $\theta$ and data *x*. Denoting the prior probability measure for $\theta$ by ${\varPi }$ with density $\pi$ with respect to a relevant support measure, the prior probability measure for a marginal parameter $\psi ={\varPsi }(\theta )$ by ${\varPi }_{{\varPsi }}$ with density $\pi _{{\varPsi }}$ and the corresponding posterior quantities by ${\varPi }_{{\varPsi }}(\cdot \,|\,x),\pi _{{\varPsi }}(\cdot \,|\,x)$, then the relative belief ratio for ${\varPsi }$ at $\psi$ is given by $RB_{{\varPsi }}(\psi \,|\,x)=\pi _{{\varPsi }}(\psi \,|\,x)/\pi _{{\varPsi }}(\psi )$ with the other quantities defined similarly and with the same interpretations. Various consistency and optimality results for these inferences are summarized in Evans (2015) and see Sect. 5.

### Model checking via relative belief

The check on the constraints should not involve the prior ${\varPi }$ since it is not involved in the production of the data. It is permissible, however, to use another prior on ${\varTheta }_{k}$ for the model checking step. This leads to formal inference methods for model checking using relative belief. The uniform prior ${\varPi }_{k}$ on ${\varTheta }_{k}$ treats all multinomials equivalently and so this prior is used for the model checking step here. Certainly alternative proper priors could be used in the model check, such as the Dirichlet$(1/2,\ldots ,1/2)$ which is Jeffreys’ prior, but a good justification for this should be provided.

Suppose first that ${\varPi }_{k}({\varTheta })>0$. The posterior probability of ${\varTheta }$ is ${\varPi }_{k}({\varTheta }\,|\,T(x))$ where ${\varPi }_{k}(\cdot \,|\,T(x))$ is the Dirichlet$(T_{1}(x)+1,\ldots ,T_{k+1}(x)+1)$ probability measure. The relative belief ratio of ${\varTheta }$ equals $RB({\varTheta }\,|\,T(x))={\varPi }_{k}({\varTheta }\,|\,T(x))/{\varPi }_{k}({\varTheta })$ and the strength of this evidence, since there are only two possibilities, can be assessed via the posterior probability ${\varPi }_{k}({\varTheta }\,|\,T(x))$. So, if $RB({\varTheta }\,|\,T(x))>1$ and ${\varPi }_{k}({\varTheta }\,|\,T(x))$ is low, there is only weak evidence in favor of ${\varTheta }$ while, if $RB({\varTheta }\,|\,T(x))<1$ and ${\varPi }_{k}({\varTheta }\,|\,T(x))$ is low, there is strong evidence against ${\varTheta }$. Similarly, if $RB({\varTheta }\,|\,T(x))>1$ and ${\varPi }_{k}({\varTheta }\,|\,T(x))$ is high, there is strong evidence in favor of ${\varTheta }$ while, if $RB({\varTheta }\,|\,T(x)) <1$ and ${\varPi }_{k}({\varTheta }\,|\,T(x))$ is high, there is only weak evidence against ${\varTheta }$.

It is often the case, however, that ${\varPi }_{k}({\varTheta })=0$, because ${\varTheta }\subset {\varTheta }_{k}$ is of lower dimension, so ${\varPi }_{k}({\varTheta }\,|\,T(x))=0$ and it is not possible to proceed as just described. If ${\varPi }_{k}({\varTheta })$ is very small, then again the preceding approach seems questionable as it cannot be expected that ${\varPi }_{k}({\varTheta }\,|\,T(x))$ will be large, and so obtain strong evidence in favor of the model, without a large amount of data. So the approach discussed in Al Labadi et al. (2017a) and Al Labadi and Evans (2018) is used in such situations. A distance measure *d* on ${\varTheta }_{k}^{2}$ is specified so $d_{{\varTheta }}(\theta )=\inf _{\theta ^{*} \in {\varTheta }}d(\theta ,\theta ^{*})$ measures the distance $\theta$ is from ${\varTheta }$ which will be denoted $d(\theta )$ hereafter as the set ${\varTheta }$ is implicit. In all the examples the distance used is $d(\theta )= \inf _{\theta ^{*}\in {\varTheta }}KL(\theta \,||\,\theta ^{*})$ where $KL(\theta \,||\,\theta ^{*})$ denotes the Kullback-Leibler divergence between the two multinomial distributions specified by $\theta$ and $\theta ^{*}$. Note that, see Pawitan (2013), the MLE of $\theta$ in the constrained model, which is the same as the relative belief estimate of $\theta$, will converge to the point in ${\varTheta }$ which minimizes the *KL* divergence between ${\varTheta }$ and the true value, which lies in ${\varTheta }_{k}$ but may be outside of ${\varTheta }$. As such, *KL* divergence seems like a natural statistical distance measure. Still alternative distance measures, such as squared Euclidean distance $d(\theta )=\inf _{\theta ^{*}\in {\varTheta }}||\theta -\theta ^{*}||^{2}$, can be used if it is felt that there is some relevance to a particular application. In general, the choice of any distance measure leads to a check on the model. In our experience, however, the results, in terms of whether evidence in favor of or against a model is obtained and the strength of that evidence, are not highly dependent on this choice.

The prior and posterior distributions of $\theta$ induce prior and posterior distributions for $d(\theta )$. If the posterior of $d(\theta )$ concentrates more (less) about 0 than the prior, then this provides evidence in favor of (against) ${\varTheta }$. The relative belief ratio given by $RB_{d} (0\,|\,T(x))=\lim _{\delta \downarrow 0}RB_{d}([0,\delta )\,|\,T(x))$ measures this as there is evidence in favor of ${\varTheta }$ when $RB_{d}(0\,|\,T(x))>1$ and evidence against when $RB_{d}(0\,|\,T(x))<1$. Typically the limit cannot be computed exactly so $\delta >0$ is selected satisfying $RB_{d}([0,\delta )\,|\,T(x))\approx \,RB_{d}(0\,|\,T(x))$. In practice, there is a $\delta >0$ such that, if $d(\theta ^{\text {true}})\in [0,\delta )$, then ${\varTheta }$ can be regarded as true or as an acceptable approximation. The value of $\delta$ can be determined by bounding the absolute error in the probabilities or bounding the relative error in the probabilities as discussed in Al Labadi et al. (2017a). The strength of the evidence can be measured by discretizing the range of the prior distribution of *d* into $[0,\delta ),[\delta ,2\delta ),\ldots$ and computing the posterior probability ${\varPi }_{k}(\{i:\,RB_{d} ([i-1)\delta ,i\delta )\,|\,T(x))\le \,RB_{d}([0,\delta )\,|\,T(x))\}\,|\,T(x))$.

The consistency of this approach to model checking follows from results in Evans (2015). As $n\rightarrow \infty$ and ${\varTheta }$ is true, the relative belief ratio converges to its maximum possible value (greater than 1) and the strength goes to 1 while when ${\varTheta }$ is false, the relative belief ratio and the strength go to 0. Note that these tests are all exact as they do not rely on asymptotics and evidence in favor of ${\varTheta }$ can be obtained.

### Computations

For all the examples considered here it is easy to generate values of $\theta$ from the uniform prior on ${\varTheta }_{k}$ and from the associated posterior. In the case where ${\varPi }_{k}({\varTheta })>0$, this leads directly to estimates of $${\varPi }_{k}({\varTheta }),{\varPi }_{k}({\varTheta }\,|\,T(x))\ $$and $RB({\varTheta }\,|\,T(x))$ based upon large Monte Carlo samples from these distributions. In cases where ${\varPi }_{k}({\varTheta })=0$ or is very small, the values of $d(\theta )$ are computed for each generated value of $\theta$ which provides large samples from the prior and posterior distributions of this quantity. As described previously, the prior and posterior densities of $d(\theta )$ are then estimated via density histograms based upon a relevant $\delta$ as determined by the application. This leads directly to computing $RB_{d}([0,\delta )\,|\,T(x))$ as the ratio of these density estimates, as well as the straightforward computation of the posterior probability giving the strength of the evidence based upon the estimated posterior density of $d(\theta )$. The Monte Carlo sample sizes used were determined by doing the computations until the results stabilized and adding some additional samples as insurance.

### Examples

Consider applications of this to the examples of Sect. 2.

#### Example 4

Goodness-of-fit for the trine model.

Table 1 contains data from two separate experiments discussed in Len et al. (2017) where two instances of the trine model are relevant and $T_{i}(x)$ is the number of clicks on the *i*-th detector.

**Table 1:** Results from two experiments based on the trine model in Example 4

	*n*	$T_{1}(x)$	$T_{2}(x)$	$T_{3}(x)$
Symmetric	7076	3416	1912	1748
Asymmetric	6756	6192	316	248

Here $${\varTheta }=\{\theta :\left( \theta -c\right) ^{t}C\left( \theta -c\right) \le 1\}$$ with

$$\begin{aligned} c=\frac{1}{2}\left( \begin{array}[c]{c} 2a\\ 1-a \end{array} \right) ,\quad C=(1-2a)^{-1}\left( \begin{array}[c]{cc} (1-1/a)^{2} &{}\quad 2\\ 2 &{}\quad 4 \end{array} \right) , \end{aligned}$$

$a=0.5\sin ^{2}(\cos ^{-1}(\cot (2\varphi _{0})))$ and $\varphi _{0}$ is an angle associated with the experiment.

For the symmetric trine $\varphi _{0}=\pi /6$ so $a=1/3$ and ${\varPi }_{3} ({\varTheta })=a\sqrt{1-2a}\pi =0.6046$. Under ${\varPi }_{3}$ the posterior distribution of $\theta$ is Dirichlet(3417, 1913, 1749) and sampling from this distribution shows that the entire posterior is concentrated within ${\varTheta }$ so $RB({\varTheta }\,|\,T(x))=1/0.6046=1.6540$. So there is evidence in favor of the symmetric trine model and this is very strong evidence since the posterior content of ${\varTheta }$ is effectively 1. For the asymmetric trine case $\varphi _{0}=2\pi /9$ so $a=0.48445$ and ${\varPi }_{3}({\varTheta })=0.2684$. Under ${\varPi }_{3}$ the posterior distribution of $\theta$ is Dirichlet(6193, 317, 249) and sampling from this distribution shows that the entire posterior is concentrated within ${\varTheta }$ so $RB({\varTheta }\,|\,T(x))=1/0.2684=3.7258$ with strength effectively 1. So there is evidence in favor of the asymmetric trine model and again this is very strong evidence. So with both models one can feel quite confident that the true values of the probabilities lie within the respective ${\varTheta }$. The evidence is definitive here because of the large amount of data.

These examples are representative of what one can expect with any of the other models listed in Example 1. Essentially these models are physically correct, at least as dictated by quantum theory, but they may not be correct based on the data collection actually carried out. In other words, if one of the physical devices was not recording clicks as it should, then the model will fail to pass the check and this is highly meaningful to the experimenter.

#### Example 5

Goodness-of-fit for the Hardy-Weinberg equilibrium model.

**Table 2:** CCR-5 genotypes in a sample of 212 aids free men in Example 5

*n*	$A_{1}A_{1}$	$A_{1}A_{2}$	$A_{2}A_{2}$
212	4	33	175

The data in Table 2 is from Laird and Lange (2011) and is concerned with CCR-5 receptor deletion which leads to protection against the HIV virus infecting certain T-cells. Let $A_{1}$ denote the dominant allele associated with deletion and $A_{2}$ denote the recessive allele. Under HWE $\theta _{11} =\omega ^{2},\theta _{12}=2\omega (1-\omega )$ and $\theta _{22} =(1-\omega )^{2}$. The probabilities corresponding to HWE comprise a set of measure 0 so the *KL* distance is used to check this model via the prior and posterior distributions of

$$\begin{aligned} d(\theta )=\inf _{\omega \in [0,1]}-\left\{ \theta _{11}\log \frac{\omega ^{2}}{\theta _{11}}+\theta _{12}\log \frac{2\omega (1-\omega )}{\theta _{12}} +\theta _{22}\log \frac{(1-\omega )^{2}}{\theta _{22}}\right\} . \end{aligned}$$

The minimizing value is $\omega =\theta _{11}+\theta _{12}/2$ so simulation from the prior/posterior distribution of $d(\theta )$ is easy.

It is necessary to specify a value for $\delta$. Suppose that, if the relative errors in the probabilities are less than $\delta$, then the HWE model is considered reasonable. So denoting the HWE probabilities by $p_{HWij}$, we want $-\delta \le (p_{HWij}-\theta _{ij})/\theta _{ij}\le \delta$ for all (*i*, *j*) for HWE to hold. Then $\log \left( p_{HWij}/\theta _{ij}\right) =\log \left( 1+(p_{HWij}-\theta _{ij})/\theta _{ij}\right) \approx (p_{HWij}- \theta _{ij})/\theta _{ij}$ when the relative error is small and so it can also be expected that $-\delta \le d(\theta )\le \delta$ whenever HWE holds and not otherwise. The *KL* distance is nonnegative so the HWE model is checked by computing $RB_{d}([0,\delta )\,|\,(T_{11}(x),T_{12}(x),T_{22}(x)))$. Using $\delta =0.01$, namely, a relative error of 1%, then $RB_{d}([0,\delta )\,|\,(T_{11}(x),T_{12}(x),T_{22}(x)))=3.43$ and the strength equals 1.00, so this is virtually categorical evidence in favor. Similarly, with $\delta =0.005$, then $RB_{d}([0,\delta )\,|\,(T_{11}(x),T_{12}(x),T_{22} (x)))=3.16$ with strength 0.64 so there is some dependence of the strength of the evidence on the relative error bound.

Laird and Lange (2011) use the chi-squared goodness-of-fit test to check the HWE model which requires an asymptotic justification for the p-value and can have inaccurate type I errors as documented in Wigginton et al. (2005) who recommend an exact permutation test. Wakefield (2010) criticizes this exact test as it can be computationally intensive and the p-values need additional calibration, so a Bayes factor approach is developed. The Bayes factor is the ratio of the posterior odds in favor of ${\varTheta }$ to the prior odds in favor and, since ${\varTheta }$ has prior probability 0 for any continuous prior, it is necessary to specify a prior probability for ${\varTheta }$ to be true, a prior distribution concentrated on ${\varTheta }$ and a prior distribution on ${\varTheta }_{k}$ (effectively on ${\varTheta }^{c}$ as ${\varTheta }$ has 0 volume), with the overall prior a discrete mixture. While the Bayes factor can be seen as a valid characterization of evidence, in the sense that it satisfies the principle of evidence also with cut-off 1, the calibration of the Bayes factor is an unresolved issue although ad hoc scales have been proposed. By contrast the approach taken here requires the specification of a single prior, which is the uniform on ${\varTheta }_{k}$, the results are exact and the relative belief ratio is calibrated via the strength. The relationship between Bayes factors and relative belief ratios is discussed in Evans (2015) where it is pointed out that the possibility of doing hypothesis assessment without the need to use a mixture prior is a distinct advantage for the relative belief approach.

#### Example 6

Goodness-of-fit for the multinomial with ordered probabilities.

A numerical example used in Izsák (2006), based on data concerned with fly diversity found in Papp (1992), is considered where the counts are given by $T(x)=(145,96,35,29,20,11,4,4,4,3,3,2,2,1,1,1,1,1)$ so $k=17$ and $n=363$. The question of interest is whether or not the data could have come from model (2), or even from the ${\text {ZM}}_{k}$ submodel. Note that testing the fit of a multinomial with ordered probabilities is quite different than testing the equality of $k+1$ binomial probabilities versus the alternative that these are ordered, as with the Cochran-Armitage test.

Consider first checking (2). For this model ${\varPi }_{k} ({\varTheta })=1/18!=1.5619\times 10^{-16}$ which is very small and so estimating ${\varPi }_{k}({\varTheta }\,|\,T(x))$ with accuracy is difficult. It is to be noted, however, that given the small prior probability of this set, if any of the values generated from the posterior for some feasible sample size fall in ${\varTheta }$, then this will give clear evidence in favor of the model. For example, in a sample of $10^{7}$ the posterior content was estimated as $10^{-7}$ and this produces a relative belief ratio of $6.4\times 10^{8}$ but the strength of this evidence in favor is extremely weak. A better approach is to group the cells into subgroups such that the hypothesized monotonicity in the model is maintained. So 9 groups can be formed by combining 2 consecutive cells or 6 groups can be formed by combining 3 consecutive cells. To select which grouping to use for the test, it makes sense to start with the finest grouping such that the posterior content can be accurately estimated but coarser groupings can also be examined. Choosing 9 groups worked as the posterior content of the relevant set was estimated as 0.0396, 0.0402 and 0.0406 based on Monte Carlo samples of sizes $10^{4},10^{5}$ and $10^{6}$, respectively. The prior content of the relevant set is $1/9!=2.755\,7\times 10^{-6}$, the relative belief ratio is 14726 but the posterior content implies only weak evidence in favor. Using groups of size 3, however, the relative belief ratio is 285.6312 and the posterior content is 0.40. So overall it is reasonable to assume (2) holds.

**Fig. 1:**
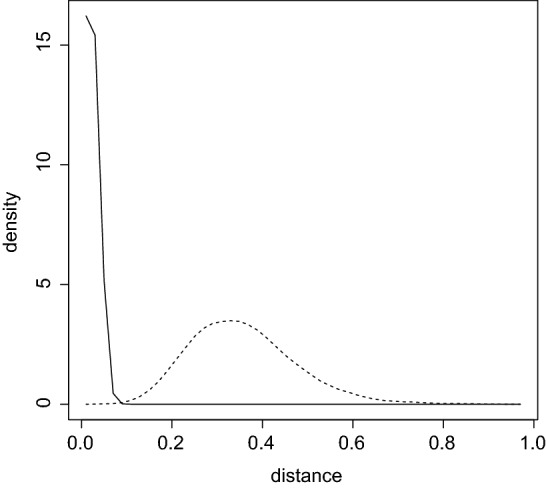
Plot of prior (- - -) and posterior (—) densities of *d* for the ${\text {ZM}}_{k}$ model in Example 6

Consider checking the ${\text {ZM}}_{k}$ model. The set of ${\text {ZM}}_{k}$ distributions has prior probability 0 with respect to ${\varPi }_{k}$ so the *KL* distance measure is used. A technical difficulty is the need to compute $d(\theta )=\inf _{\alpha >-1,\beta \ge 0}\sum _{i=1}^{k+1} \theta _{i}\ln (\theta _{i}C_{k}\left( \alpha ,\beta \right) (\alpha +i)^{\beta })$ for each generated $\theta$, to obtain samples from the prior and posterior distribution of *d*. For this a large table of ${\text {ZM}}_{k}$ distributions was created, $d(\theta )$ computed for each element and the minimum value found. There is a redundancy in the parameterization as, for example, uniformity is well-approximated by many values of $\left( \alpha ,\beta \right)$. As in Example 5, a value of $\delta >0$ was selected so that if the *KL* distance between two distributions is less than $\delta$, then this difference is irrelevant. For any $\beta \ge 0$, it is only necessary to consider values of $\alpha$ such that the *KL* distance is greater than or equal to $\delta$ which places an upper bound on $\alpha$. Figure 1 is plot of the prior and posterior densities of *d* based on Monte Carlo samples of size $10^{5}$, using $\delta =0.02$ and some smoothing. It is clear that the posterior has become much more concentrated about the ${\text {ZM}}_{k}$ model than the prior. Furthermore, $RB_{d}(([0,\delta )\,|\,f)=1.75\times 10^{3}$ and the strength equals 1. So there is ample evidence in favor of the ${\text {ZM}}_{k}$ model. This agrees somewhat with the finding in Izsák (2006) who conducted a goodness-of-fit test via computing a p-value based on the chi-squared statistic after grouping and found no evidence against the model. With the methodology developed here, however, there is no need to appeal to asymptotics and evidence in favor of the model has been found.

## Checking the prior

Suppose the model has been accepted and attention now focuses on the prior ${\varPi }$. A prior is inappropriate when the prior places relatively little mass in a region of the parameter space containing the true value of the model parameter. The true value is not known but it is still possible to say something about this through the data. The approach taken in Evans and Moshonov (2006) is used here and involves comparing the observed value of a statistic with its prior predictive distribution through a certain tail probability which, when small, indicates that a prior-data conflict exists which in turn suggests that the prior is not putting its mass in the right place. For the contexts considered here, with prior ${\varPi }$ having density $\pi$ on ${\varTheta }$, the prior predictive density of the cell counts $T=(T_{1} ,\ldots ,T_{k+1})$, evaluated at ${\mathbf {t}}=(t_{1},\ldots ,t_{k+1})$ where $t_{i}\in {\mathbb {N}}_{0}$ and $t_{1}+\cdots +t_{k+1}=n$, is given by the probability function $$m_{T}({\mathbf {t}})=\left( {\begin{array}{c}n\\ t_{1}\ldots t_{k+1}\end{array}}\right) \int _{{\varTheta }} \prod _{j=1}^{k+1}\theta _{j}^{t_{j}}\,\pi (\theta )\,d\theta $$. The prior predictive probability measure associated with this probability function is then denoted by $M_{T}$ and the tail probability is

5 $$\begin{aligned} M_{T}(m_{T}({\mathbf {t}})\le m_{T}(T(x))). \end{aligned}$$

The tail probability (5) is measuring where the observed *T*(*x*) lies with respect to its prior distribution. If (5) is small, then *T*(*x*) lies in a region of low prior probability and the data and the prior are in conflict. The check on the prior should depend on the data only through *T*(*x*) because the conditional distribution of the data *x* given *T*(*x*), does not depend on $\theta$ and so implies nothing about the prior.

In Evans and Jang (2011a) it is proved that, under fairly general conditions, (5) converges to ${\varPi }(\pi (\theta )\le \pi (\theta ^{\text {true}})$ as $n\rightarrow \infty$. This supports the assertion that a small value of (5) is indicating that the prior is placing its mass in the wrong place. Note, however, that it is the discrepancy between the observed value *T*(*x*) and its prior distribution that leads to the conclusion that a prior-data conflict exists. So the goal here is to see if such a conflict exists and the fact that (5) is consistent estimator of ${\varPi }(\pi (\theta )\le \pi (\theta ^{\text {true}})$ suggests that this check is doing the right thing. In other words, our purpose is not to necessarily estimate ${\varPi }(\pi (\theta )\le \pi (\theta ^{\text {true}})$. One of the conditions for convergence is that $M_{T}$ be continuous and here $M_{T}$ is always discrete. It was proved in Evans and Jang (2011a), however, that when $k=1$ a modified version of $M_{T}$ can be constructed that yields the consistency result in the binomial case and this is described in the “Appendix”. The following much more difficult result is proved in the “Appendix” for the constrained multinomial where ${\varTheta }_{k,c},{\varTheta }_{k,d}$ denote the sets of continuity and discontinuity points of $\pi$.

### Theorem 1

Let $\pi$ be a prior on $\theta \in {\varTheta }_{k}$ that satisfies the following conditions. (A1) The prior density is bounded above, that is, there exists $B>0$ such that $\pi (\theta )\le B$ for all $\theta \in {\varTheta }_{k}$. (A2) The prior density is continuous almost surely with respect to volume measure, that is, $\text {vol}({\varTheta }_{k,d})=0$. (A3) The prior probability of each level set of prior density is a null set with respect to the prior, that is, ${\varPi }(\{\theta :\pi (\theta )=l\})=0$ for any $l\ge 0$. Then (5) converges to ${\varPi }(\pi (\theta )\le \pi (\theta ^{\text {true}}))$ whenever $\theta ^{\text {true}}\in {\varTheta }_{k,c}$ as $n\rightarrow \infty$.

The proof also establishes a generalization.

### Corollary 1

If $\pi$ satisfies (A1) and (A2) and is continuous at $\theta ^{\text {true}}$, then ${\varPi }(\pi (\theta )<\pi (\theta ^{\text {true}}))\le \liminf _{n\rightarrow \infty }M_{T} (m_{T}({\mathbf {t}})\le m_{T}(T(x)))\le \limsup _{n\rightarrow \infty }M_{T}(m_{T}({\mathbf {t}})\le m_{T}(T(x))) \le {\varPi }(\pi (\theta )\le \pi (\theta ^{\text {true} }))$.

So, if ${\varPi }(\pi (\theta )<\pi (\theta ^{\text {true}}))$ is large or if ${\varPi }(\pi (\theta )\le \pi (\theta ^{\text {true}}))$ is small, then $M_{T} (m_{T}({\mathbf {t}}) \le m_{T}(T(x)))$ should reflect this even without A3.

The value (5) is invariant under 1-1 transformations of *T* and under reparametrizations. So, if we reparameterize and use the transformed prior to compute $m_{T}$, this has no effect on the limit. The theorem requires ${\varPi }$ to be a continuous probability measure and $\pi$ cannot be constant on a subregion of ${\varTheta }_{k}$ having positive volume. For example, the theorem does not cover the uniform prior on ${\varTheta }_{k}$ but the result still holds in this case since (5) and ${\varPi }_{k}(\pi (\theta )\le \pi (\theta ^{\text {true}}))$ both equal 1.

Given that the prior, like the sampling model, is a subjective choice, it is a natural scientific requirement that it should be subjected to a check against the data as all ingredients to a statistical analysis should be falsifiable and subjected to such a test. As discussed in Al Labadi and Evans (2017b), the existence of a prior-data conflict can lead to inferences that are sensitive to small changes in the prior and so a check on the prior can be viewed as a way of assessing sensitivity to the prior although such sensitivity can also arise when there is no conflict simply because of a small amount of data. When such sensitivity exists, the prior can be thought of as having too much influence on the inferences as opposed to the data. Also, the correct way to choose a prior for a problem is through elicitation where an expert translates background knowledge about the context in question into a prior probability distribution. The existence of a prior-data conflict, then indicates that there either something wrong with the elicitation process or with the knowledge the expert has expressed. Especially when a default proper prior is chosen, as is sometimes done in studies concerning sparsity, it is important that the prior be subjected to such a check to make sure the choice is not contradicted by the data. In any case something valuable has been learned after such a check has been conducted. More broadly speaking checking for prior-data conflict is about the process, as with model checking, whereby one argues that a particular statistical analysis is justified.

A natural question to consider is what is to be done when a prior is found to be in conflict. A similar question can be asked about the model when it fails its checks. For the prior, Evans and Jang (2011b) consider what it means for one prior to be weakly informative with respect to another and the definition involves specifying mathematically that the more weakly informative prior will result in fewer prior-data conflicts and this is expressed via a precise percentage. So before the data is obtained, one can specify a hierarchy of progressively more weakly informative priors starting from a base elicited prior, and if a prior-data conflict is obtained, then move up the hierarchy until this is avoided. Since the hierarchy of priors is specified before seeing the data, the final selected prior is not strongly dependent on the data. The downside of this approach is that it can be complicated to specify the hierarchy and that is certainly the case for constrained multinomials and so this problem is not pursued further here. Often, however, as with the Examples, intuition works to specify a more weakly informative prior, but this is not always the case as demonstrated in Evans and Jang (2011b).

### Computations

In general the computation of (5) can be difficult but a high degree of precision is not essential for the purpose of checking the prior. For this computation an algorithm to generate ${\mathbf {t}}\sim M_{T}$ is needed and this can often be accomplished by generating $\theta \sim {\varPi }$, then generating ${\mathbf {t}}\sim$ multinomial$(n,\theta _{1},\ldots ,\theta _{k+1})$, which is the case for all the examples considered here. If $m_{T} ({\mathbf {t}})$ is available in closed form (Example 8), then it is straightforward to estimate (5) via a large Monte Carlo sample from $M_{T}$ and checking the inequality in (5) for each generated ${\mathbf {t}}$. If $m_{T}({\mathbf {t}})$ is not available in closed form (Examples 7 and 9), then it is necessary to approximate the integral $\int _{{\varTheta }} \prod _{j=1}^{k+1}\theta _{j}^{t_{j}}\,\pi (\theta )\,d\theta$. As discussed in these examples, importance sampling can be used to estimate the integral in an inner Monte Carlo loop, for each generated ${\mathbf {t}}$ as well as for *T*(*x*).

In some examples a parameterization of the Dirichlet$(\alpha _{1},\ldots ,\alpha _{k+1})$ distribution with all $\alpha _{i}>1$ will be used given by the mode $(\xi _{1},\ldots ,\xi _{k+1})$, where $\xi _{i} =(\alpha _{i}-1)/\tau$, and the *concentration parameter* $\tau =\alpha _{1}+\cdots +\alpha _{k+1}-(k+1)$. As $\alpha _{i}=1+\tau \xi _{i}$, the set of all Dirichlets with this mode is indexed by $\tau >0$. The mean and variance of the *i*-th coordinate equal $(1+\tau \xi _{i})/(\tau +k+1)$ and $(1+\tau \xi _{i})(\tau +k-\tau \xi _{i}))/(\tau +k+1)^{2}(\tau +k+2)$ which converge respectively to $\xi _{i}$ and 0 as $\tau \rightarrow \infty$, so the distribution concentrates at the mode. As $\tau \rightarrow 0$, the distribution converges to the uniform on ${\varTheta }_{k}$.

### Examples

Consider now the examples of Sect. 2.

#### Example 7

Checking the prior for the trine.

For a single qubit, an experimenter without any prior knowledge could assign a prior to the qubit state space that is uniform under the Hilbert-Schmidt measure. When a trine measurement is performed on the qubit, this results in the prior given by $\pi (\theta )\varpropto (1-\left( \theta -c\right) ^{t}C\left( \theta -c\right) )^{1/2}$ when $\theta \in {\varTheta }$ and 0 otherwise, where *c* and *C* are as in Example 4. The transformation $\theta \rightarrow (r,\omega )$, where $\theta =c+C^{1/2}r^{1/2}(\cos \omega ,\sin \omega )^{t}$ gives $\omega \sim U(0,2\pi )$ independent of $r\sim$ beta(3/2, 3/2) which provides an algorithm for generating from $\pi$. When *n* is modest, generating from $\pi$ and averaging the likelihood can be used to compute the values $m_{T}(t_{1},t_{2},t_{3})$ needed for (5). In this case *n* is large so this is too inefficient due to the concentration of the likelihood near the MLE over ${\varTheta }_{k}$. The posterior under the uniform prior on ${\varTheta }_{k}$ also concentrates near the MLE and so importance sampling based on sampling from the Dirichlet$(t_{1}+1,t_{2}+1,t_{3}+1)$ is used to estimate $m_{T}(t_{1},t_{2},t_{3})$ for each $(t_{1},t_{2},t_{3})$ in a sample drawn from $m_{T}$. Sampling from $m_{T}$ is carried out by generating $(\theta _{1},\theta _{2},\theta _{3})$ from $\pi$ and then generating $(t_{1},t_{2},t_{3})\sim$ multinomial$(n,\theta _{1},\theta _{2},\theta _{3})$. The values of $m_{T}(t_{1},t_{2},t_{3})$ are compared to $m_{T}(T_{1} (x),T_{2}(x),T_{3}(x))$ to estimate (5).

This procedure was carried out for the entries in Table 1 with $10^{3}$ values of $(t_{1},t_{2},t_{3})$ generated from $m_{T}$ and each value of $m_{T} (t_{1},t_{2},t_{3})$ estimated using a sample of $10^{4}$ from the relevant posterior based on $(t_{1},t_{2},t_{3})$. Prior-data conflict in this example corresponds to the true value of $\theta$ lying near the boundary of the respective set ${\varTheta }$. For the symmetric case (5) was estimated as 0.87 and for the asymmetric case (5) was estimated as 0.15. These results were quite stable over different choices of simulation sample sizes. So in neither case is there any indication of prior-data conflict.

#### Example 8

Eliciting and checking the prior for the HWE model.

Consider first a Dirichlet$(\alpha _{1},\ldots ,\alpha _{l})$ prior on the probabilities for the alleles $\omega =(\omega _{1},\ldots ,\omega _{l})$. There are variety of approaches to eliciting the $\alpha _{i}$ values, see Elfadaly and Garthwaite (2013, 2017), but a method discussed in Evans, Guttman and Li (2017) is used here. This is based on prescribing a lower bound $0\le a_{i}\le \omega _{i}$ or an upper bound $1\ge b_{i}\ge \omega _{i}$ on each probability $\omega _{i}$ such that these bounds hold simultaneously with at least some prescribed prior probability $\gamma$. If all lower bounds $a_{i}$ are specified, then the upper bounds $\omega _{i}\le b_{i}=1-\sum _{j\ne i}a_{j}$ are immediate and of course it is required that the lower bounds satisfy $\sum _{j=1}^{l}a_{j}\le 1$ which is easily satisfied by weakening some of the bounds. Similar results are available if all upper bounds or a mixture of lower and upper bounds are specified. Such bounds specify a subsimplex of the full simplex of probabilities, with all edges of the same length, and so it takes into account the dependencies among the $\omega _{i}$. It is also required that the $\alpha _{i}\ge 1$, to avoid singularities on the boundaries. So specifying the mode, for example as the centroid of the subsimplex, and then choosing $\tau$ so that the subsimplex contains $\gamma$ of the prior probability, fully specifies the prior. It is natural to take $\gamma$ large, say $\gamma \approx 0.99$, to reflect the fact that one is ‘virtually certain’ that the bounds hold but still allow for the possibility that they don’t.

It also seems reasonable that one could place such bounds on the probabilities for the genotypes. This requires $c_{i}$ satisfying $0\le c_{i}\le \omega _{i}^{2}$ and $(\sum _{i=1}^{l}c_{i}^{1/2})^{2}\le 1$. Since this constraint also implies $\sum _{i=1}^{l}c_{i}^{1/2}\le 1$, this is equivalent to the previous approach but with $a_{i}=c_{i}^{1/2}$. Again it is possible to have a mixture of upper and lower bounds.

A closed form expression is available for $m_{T}$ as this is the expectation of a polynomial in $\omega$ with respect to a Dirichlet distribution. Then (5) is computed by generating $\omega \sim {\varPi }$, generating *t* from the relevant multinomial, computing $\log m_{T}(t)$ and comparing this to $\log m_{T}(T(x))$, and doing this multiple times.

In the case of the CCR-5 receptor deletion data of Example 5, $l=2$ and so we specify $a_{1}\le \omega _{1}$ and $a_{2}\le 1-\omega _{1}$ which implies $a_{1}\le \omega _{1}\le 1-a_{2}$. With $(\xi _{1},\xi _{2})=((a_{1} +1-a_{2})/2,(a_{2}+1-a_{1})/2)$, then $(\alpha _{1},\alpha _{2})=(1,1)+\tau (\xi _{1},\xi _{2})$ so $\tau$ is chosen so that the beta$(1+\tau \xi _{1} ,1+\tau \xi _{2})$ distribution has $\gamma$ of its probability in the interval $(a_{1},1-a_{2})\subset [0,1]$. For illustrative purposes, suppose $a_{1}=0.1$ and $$a_{2}=0.5\ $$so $(\xi _{1},\xi _{2})=(0.3,0.7)$ and then $\tau =34.5$ gives that the beta(11.35, 25.15) distribution assigns probability 0.99 to (0.1, 0.5). This leads to $m_{T}(4,33,175)=7.407\times 10^{-6}$ and (5) equal to 0.005 indicating that a prior-data conflict exists. So these bounds are inappropriate and indeed we would expect a geneticist to be able to state more realistic bounds for either the allele probabilities or the genotype probabilities for a particular population. A more weakly informative prior on $\omega _{1}$ will avoid prior-data conflict. For example, the bounds $a_{1}=0.01,a_{2}=0.7$ lead to $\tau =53$ and a beta(9.215, 45.785) prior on $\omega _{1}$, which assigns 0.99 of its probability to (0.01, 0.30), and then (5) equals 0.180 and there is no prior-data conflict in this case. Of course, one could also use a “noninformative” uniform(0, 1) prior on $\omega _{1}$ and then (5) equals 0.241, indicating no prior-data conflict. The recommendation, however, is that an elicited informative prior always be used when this is possible and always be checked for prior-data conflict. The use of a default prior suggests that little thought relevant to the application has gone into the choice of the prior. While what seems like a natural noninformative prior exists for $\omega _{1}$ in this model, this is not the case in general.

#### Example 9

Eliciting and checking the prior for the multinomial with ordered probabilities.

It is necessary to provide an elicitation algorithm for a prior on $\theta$ satisfying (2). For this Lemma 1 helps considerably since (3) implies that any prior on $\omega$ induces a prior on $\theta$ satisfying (2). It is natural to choose a Dirichlet$(\alpha _{1}, \ldots ,\alpha _{k+1})$ prior on $\omega$ but how should the $\alpha _{i}$ be chosen? This of course depends upon what is known about the $\theta _{i}$. One approach is to specify ordered probabilities $\theta _{1}^{*}\ge \theta _{2}^{*}\ge \cdots \ge \theta _{k+1}^{*}$ and then use a prior with mode at this point. By Lemma 1 this can be accomplished by a Dirichlet prior on $(\omega _{1},\ldots ,\omega _{k+1})$ with mode at $(\xi _{1},\ldots , \xi _{k+1})^{\prime }=A_{k}^{-1}(\theta _{1}^{*},\ldots ,\theta _{k+1}^{*})^{t}$ and the concentration $\tau$ can be chosen to reflect the degree of belief in this mode. This requires that the $\theta _{i}^{*}$ also comprise a probability distribution. The following result characterizes the choices of $\theta _{i}^{*}$ that are equispaced as this seems like a reasonable choice although there are many other possibilities that can be characterized similarly.

#### Lemma 2

The probabilities $\theta _{i}^{*}$ satisfying (2) are equispaced with $\theta _{i}^{*}=\theta _{1}^{*}-(i-1)\varepsilon$ iff $\theta _{1}^{*}=k\varepsilon /2+1/(k+1)$ and $0\le \varepsilon \le 2/k(k+1)$ and in this case $\xi _{i}=i\varepsilon$ for $i=1,\ldots ,k$ and $\xi _{k+1}=1-k(k+1)\varepsilon /2$. Then the prior is $(\omega _{1},\ldots ,\omega _{k+1})\sim$ Dirichlet$(1+\tau \varepsilon ,\ldots ,1+k\tau \varepsilon ,1+\tau (1-k(k+1)\varepsilon /2))$.

#### Proof

By Lemma 1 the $\theta _{i}^{*}=\theta _{1}^{*} -(i-1)\varepsilon$ give probabilities satisfying (2) for some $\varepsilon \ge 0$ iff $\xi _{i}=i\varepsilon$ for $i=1,\ldots ,k$ and $\xi _{k+1}=\left( k+1\right) (\theta _{1}^{*}-k\varepsilon )$ and $\theta _{1}^{*}\ge k\varepsilon$. Since $1=\sum _{j=1}^{k+1}\xi _{j} =k(k+1)\delta /2+\left( k+1\right) (\theta _{1}^{*}-k\delta )=\left( k+1\right) (\theta _{1}^{*}-k\delta /2)$ iff $\theta _{1}^{*} =k\delta /2+1/(k+1)$ and this satisfies $\theta _{1}^{*}\ge k\delta$ iff $\delta \le 2/k\left( k+1\right)$. $\square$

It remains to determine $\tau$. For this prescribe an interval (*l*, *u*) such that it is believed all the true probabilities lie in (*l*, *u*) with virtual certainty, namely,

6 $$\begin{aligned} \gamma \le {\varPi }(l<\theta _{k+1},\theta _{1}<u)={\varPi }(l<\omega _{k+1}/(k+1), {\textstyle \sum \nolimits _{i=1}^{k+1}} \omega _{i}/i<u) \end{aligned}$$

where $\gamma$ is a large probability like 0.99. Since $l<\theta _{k+1}^{*} \le \theta _{1}^{*}<u$, the right-hand side of (6) goes to 1 as $\tau \rightarrow \infty$. Therefore, $\tau$ satisfying (6) is easily found by simulation and the smallest such value of $\tau$ implies the least concentration for the prior.

**Fig. 2:**
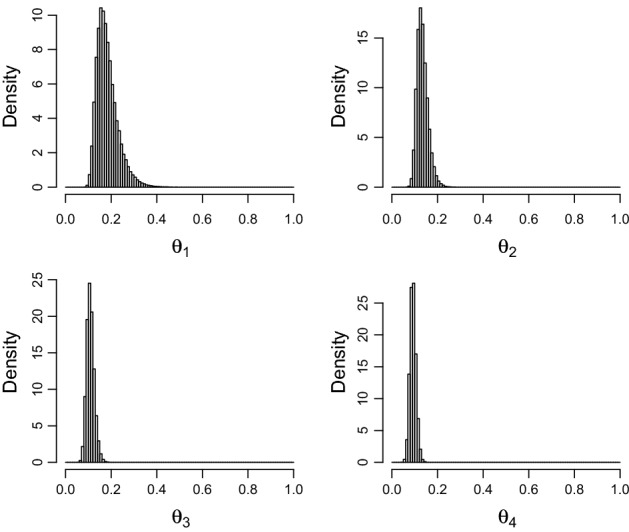
The marginal priors on $\theta _{1},\theta _{2},\theta _{3},\theta _{4}$ when $\varepsilon =0,l=1/450,u=1/2$ and $\tau =2.85$ in Example 9

For the data of Example 6 with $\varepsilon =0$, then $\theta _{1}^{*} =\theta _{k+1}^{*}=1/18$, and with $l=1/450,u=1/2$ as bounds, the value $\tau =2.85$ is the estimated smallest value satisfying (6) with $\gamma =0.99$. When $\varepsilon =2/k\left( k+1)\right)$, then $\theta _{1}^{*}=1/9$ and $\theta _{k+1}^{*}=0$ so $l=0,u=1/4$ are possible and the value $\tau =16.5$ is the estimated smallest value satisfying (6) with $\gamma =0.99$. Now consider checking the prior corresponding to $\varepsilon =0$ and $l=1/450,u=1/2$. Figure 2 is a plot of density histograms for the first four probabilities based on a sample of $10^{5}$ from the full prior.

Our approach to computing (5) is via importance sampling. For this particular data set, $$(T_{1}(x)/n,\ldots ,T_{18}(x)/n)\in {\varTheta }$$, an importance sampler on $${\varTheta }$$ with this mode and values of $$\tau \approx 60$$ produces stable estimates of $$m_{T}(T_{1}(x),\ldots ,T_{18}(x))$$. A problem occurs, however, when computing other values of $$m_{T}({\mathbf {t}})$$ necessary for estimating (5). Values of $${\mathbf {t}}$$ are obtained by generating $$(\theta _{1},\ldots ,\theta _{18})\sim \pi $$ and $$(t_{1},\ldots ,t_{18})\sim $$ multinomial$$(n,\theta _{1},\ldots ,\theta _{18})$$. When *n* is not large relative to dimension, as is the case here, then typically $$(t_{1}/n,\ldots ,t_{18}/n)\notin {\varTheta }$$ and so the choice of importance sampler is unclear. A solution is to use an importance sampler based on Lemma 1 where the Dirichlet on $$(\omega _{1},\ldots ,\omega _{k+1})$$ has its mode at $$A^{-1}(\theta _{1}^{**}, \ldots ,\theta _{18}^{**})^{\prime }$$ where $$(\theta _{1}^{**}, \ldots ,\theta _{18}^{**})$$ is the convex combination of the prior mode and $$(t_{1}/n,\ldots ,t_{18}/n)$$ that just satisfies being in $$\partial {\varTheta }$$, the boundary of the region $${\varTheta }$$. This always generates points inside $${\varTheta }$$ and should at least somewhat mimic the integrand over $${\varTheta }$$ provided the concentration $$\tau $$ is not chosen too large or too small. Here $$\tau $$ was chosen such that both smaller and larger values led to smaller estimates of (5). When this was carried out on this example the value of (5) was estimated as 0.36 which indicates there is no prior-data conflict. This makes sense as the naive estimate $$(T_{1} (x)/n,\ldots ,T_{18}(x)/n)$$ is not only in $${\varTheta }$$ but also satisfies the bounds. Suppose instead the data $$T(x)=(35,29,20,145,96,11,4,4,4,3,3,2,2,1,1,1,1,1)$$ was observed that clearly violates the monotonicity. In this case (5) equals 0 and so prior-data conflict was detected as is correct. Also, with the original data and the prior determined by $$l=0,u=0.2$$ satisfying (6), then (5) equals 0 again indicating a definite conflict.

A general approach to the computational problem is to reduce dimension by grouping as in Example 6. Intuitively, as the ratio of dimension to sample size decreases, the values of the generated relative frequencies are more likely to be in ${\varTheta }$ as confirmed by a simulation experiment. Implementing the importance sampling for the reduced problem, however, requires the computation of the marginal prior density and this is not in closed form. Since this would be required at each generated value from the importance sampler, the computational advantage is largely negated. A modification of this is based on the elicitation algorithm and could be called *marginalizing the elicitation*. Consider instead being presented with the reduced problem and applying the elicitation algorithm to it. This will not result in a prior that is the marginal of the full prior but checking this prior for conflict with the data is also assessing whether or not the information being used to choose the prior is appropriate. For example, the original elicitation led to the inequality $u\ge \theta _{1}\ge \cdots \ge \theta _{k+1}\ge l$ holding with virtual certainty and recall that necessarily $\theta _{i}\le 1/i$. So, if cells are grouped in pairs to maintain the monotonicity and to make the best use of the bounds, then supposing $k+1$ is even, $u+1/(1+(k+1)/2)\ge \theta _{1}+\theta _{1+(k+1)/2}\ge \theta _{2} +\theta _{2+(k+1)/2}\ge \cdots \ge \theta _{(k+1)/2}+\theta _{k+1}\ge l+1/(k+1)$. If $k+1$ is odd, then the last group can consist of $\theta _{k+1}$ by itself and the lower bound doesn’t change. So for even modest *k* the bounds will not increase by much and clearly this idea can be extended to groups of 3, 4 etc. Lemma 1 can then be used to obtain the prior for the parameters for the grouped problem. Supposing there are *m* groups and $(t_{1}^{\text {red}},\ldots ,t_{m}^{\text {red}})$ denotes a value generated from the prior predictive for the reduced problem, our recommendation is that this reduction be continued until a reasonable proportion of the values $(t_{1}^{\text {red}}/n,\ldots ,t_{m}^{\text {red}}/n)$ lie in the reduced parameter space. When this is the case even those that lie outside should be close to the relevant set which will improve the quality of the importance sampling. Since the model has been accepted, however, it seems likely that any points outside will still be close to ${\varTheta }$. For $10^{4}$ values of $(t_{1}^{\text {red}}/n,\ldots ,t_{m}^{\text {red}}/n)$ generated from the prior, the following values of $(m,p_{m})$ were obtained where $p_{m}$ is the proportion that lay inside the relevant parameter space: (18, 0.00), (9, 0.04), (6, 0.25), (5, 0.44), (4, 0.61), (3, 0.78) and (2, 0.93). The values 0.41 and 0.55 were obtained for (5) when $m=9$ and $m=6$, respectively. So one can feel fairly confident that the elicited information is not in conflict with the data.

The ${\text {ZM}}_{k}$ model was also considered but a significant problem remains unresolved. It is unclear how to elicit a prior on $\left( \alpha ,\beta \right)$ as the interpretation of these parameters is not obvious. The ${\text {ZM}}_{k}$ family also imposes sharper constraints on the probabilities. For example, the maximum probabilities over all $\left( \alpha ,\beta \right)$ for $i=1,2,3,4$ are 1.00, 0.33, 0.26, 0.25, respectively, which contrasts with 1.00, 0.50, 0.33, 0.25 for the general model for ordered probabilities. Since the general model for ordered probabilities is easier to use and interpret, it is recommended over the ${\text {ZM}}_{k}$ model.

## Performance measurement

It is reasonable to ask how the model checking and checking for prior-data conflict approaches discussed here perform relative to alternatives. For checking for prior-data conflict, the only approach for which consistency results exist is, as far as we know, the one described here. While simulation results can lead to insight, nothing definitive can be concluded by such an approach to making comparisons with other methods for detecting prior-data conflict. The use of (5) has been considered in many contexts, as discussed in Evans and Moshonov (2006), with favorable results both when the prior can be considered as appropriate and otherwise. So there is no hesitancy in recommending the check for prior-data conflict discussed here.

More can said about the model checking method and this is because optimality results have been established for relative belief inferences generally. For example, consider the probabilities

7 $$\begin{aligned} M_{k,T}(RB_{d}(0\,|\,{\mathbf {t}})>1\,|\,d(\theta )=d_{0})\text { and } M_{k,T}(RB_{d}(0\,|\,{\mathbf {t}})<1\,|\,d(\theta )=0) \end{aligned}$$

where $M_{k,T}(\cdot \,|\,d(\theta )=d_{0})$ denotes the conditional prior predictive measure for *T* given that $d(\theta )=d_{0}$ and the probability function $m_{k,T}({\mathbf {t}}\,|\,d(\theta )=d_{0})$ is obtained as described previously for $m_{T}({\mathbf {t}})$ based on prior $\pi$, but now the integration is with respect to the conditional prior $\pi _{k}(\cdot \,|\,d(\theta )=d_{0})$. So these conditional prior predictive distributions are now obtained using the prior ${\varPi }_{k}$, appropriately conditioned, that was used for model checking. When $d_{0}>0$ the first probability in (7) is the prior probability of obtaining evidence in favor of the model given that the model is false at a specific distance and the second is the prior probability of obtaining evidence against the model given that the model is true. Put $A_{RB}=\{\mathbf {t:}RB_{d}(0\,|\,{\mathbf {t}})>1\}$ and $R_{RB}=\{\mathbf {t:}RB_{d}(0\,|\,{\mathbf {t}})<1\}$ so $A_{RB}$ can be considered as an acceptance region for the model and $R_{RB}$ can be considered as a rejection region for the model. Applying Proposition 4.7.9 of Evans (2015) implies that, among all acceptance regions *A* satisfying $M_{k,T}(A\,|\,d(\theta )=0)\ge M_{k,T}(A_{RB}\,|\,d(\theta )=0)$, then $A_{RB}$ minimizes the prior probability of accepting the model given that it is false and, among all rejection regions satisfying $M_{k,T}(R\,|\,d(\theta )=0)\le M_{k,T}(R_{RB}\,|\,d(\theta )=0)$, then $R_{RB}$ maximizes the prior probability of rejecting the model when it is false. Actually a fair comparison demands that there be equality $M_{k,T}(A\,|\,d(\theta )=0)=M_{k,T}(A_{RB}\,|\,d(\theta )=0)$ and $M_{k,T}(R\,|\,d(\theta )=0)=M_{k,T}(R_{RB}\,|\,d(\theta )=0)$ and then it is seen that the relative belief approach to determining evidence is clearly optimal. These conclusions depend on the measure of distance *d* used, but basing this on KL divergence is a natural choice.

These results are similar to the Neyman-Pearson formulation for hypothesis testing problems. Some intuition for these results can be seen from the Savage-Dickey ratio result which implies $RB_{d}(d_{0}\,|\,T(x))=m_{k,T} (T(x)\,|\,d(\theta )=d_{0})/m_{k,T}(T(x)\mathbf {)}$, see Evans (2015). Also, Evans and Guo (2019) establishes close links between relative belief inferences and the ability to satisfy frequentist criteria. Of course, the general optimality results depend on the model and prior being correct but here there is no prior-data conflict from the uniform prior ${\varPi }_{k}$ and some specific multinomial holds provided the sampling is *i*.*i*.*d*. So these results represent strong support for the model checking approach taken here. Many other good properties have been established for relative belief inferences and these are summarized in Evans (2015).

The consistency of relative belief inferences has been established in Evans (2015). So, if the model is wrong, the relative belief ratio will converge to 0 as well as the posterior probability used to measure the strength, indicating maximally strong evidence against. Similarly, when the model is correct the relative belief ratio converges to the maximal possible value and the strength converges to 1, indicating maximally strong evidence in favor. So the only real issue is whether there is enough data available in a problem to detect model failure or avoid such a finding when it is not appropriate. This in turn is dependent on the dimension of the model and, when the sample size is too low relative to the dimension, then problems can ensue, as in Example 6, and some modifications are in order. Currently there is no general result prescribing appropriate sample sizes for a constrained multinomial.

## Conclusions

Constrained multinomial models arise in a number of interesting contexts and pose some unique challenges. A model checking procedure that allows for evidence in favor of as well as evidence against a constrained multinomial has been presented. A significant consistency theorem has been established for the check on the prior. As a particular application, a general model for ordered probabilities has been developed, together with an elicitation algorithm for a prior, and the results applied. Constrained multinomial models from quantum state estimation and genetics have also been discussed as applications.

There are many other models that correspond to constraints placed upon a multinomial. For example, the papers Iliopoulos et al. (2007) and Demirhan (2013) discuss Goodman’s RC model and generalizations of this as well as various constrained loglinear models. The approach discussed in this paper can be applied to check these models although in some cases there may be computational issues that need to be resolved depending on the distance measure *d* employed. In general, the primary issue in implementing the model check is the need to compute the distance $d(\theta )$ which can be difficult when the region ${\varTheta }$ is complex. In such contexts it may be necessary to simplify the problem somewhat by using some lower dimensional characteristic $\psi ={\varPsi }(\theta )$ such that the distance of $\psi$, for an unrestricted value of $\theta$, to the restricted set of such values can be computed. While this is not the full formal model check described in the paper, this will still serve as a check. Such issues arise with some higher dimensional quantum state models and at present there is no general solution to propose for these. It is to be noted that model checks can take a variety of forms with there being no absolutely correct approach.

The computations associated with checking the prior are, as exemplified by several of the examples, more difficult. The primary issue there is the evaluation of the prior predictive density $m_{T}$ which can be difficult to compute depending on the choice of the prior. When there is a convenient algorithm for $m_{T}$, then implementation is often straightforward although, as exemplified by Example 9 this can be difficult if the dimension of $\theta$ is high relative to the amount of data. As carried out in that example, it can be necessary to make some modifications to the full formal check in such cases as in choosing a characteristic $\psi ={\varPsi }(\theta )$ for which the induced prior is computationally tractable and then checking that prior for prior-data conflict.

The R programs associated with the examples discussed here are available at: http://utstat.utoronto.ca/mikevans/software/Constrainedmultinomial/

## Appendix

*Binomial Case* The result for the binomial, established in Evans and Jang (2011), is stated here. It is necessary to first state the general result proved there for *i*.*i*.*d*. sampling from some model. Suppose that $S_{n}$ is an estimator of the model parameter $\theta$ with observed value $S_{n}(x_{n})$, where $x_{n}$ denotes a sample of size *n*. Typically $S_{n}$ is a function of the minimal sufficient statistic and note that it takes its values in the parameter space ${\varTheta }$. It is established (Theorem 1 of that paper) that, as $n\rightarrow \infty$,

8 $$\begin{aligned} M_{S_{n}}(m_{S_{n}}(s)\le m_{S_{n}}(S_{n}(x_{n})))\rightarrow {\varPi }(\pi (\theta )\le \pi (\theta ^{\text {true}})) \end{aligned}$$

provided that ${\varTheta }$ is an open subset of a Euclidean space, (i) $S_{n}(x_{n})\rightarrow \theta$ almost surely with respect to the sampling distribution given by $\theta$ (ii) $m_{S_{n}}(s)\rightarrow \pi (s)$ uniformly on compact subsets of ${\varTheta }$ and (iii) $\pi$ is continuous and the prior distribution of $\pi (\theta )$ has no atoms. In the case of sampling from the binomial $S_{n}(x_{n})={\bar{x}}_{n}$, then (ii) does not apply because $\bar{x}_{n}$ takes its values on the lattice $\{0,1/n,\ldots ,(n-1)/n,1\}$ and $m_{S_{n}}(s)=0$ whenever *s* is not a lattice point. Note that the length measure of the set of nonlattice points is 1 for every *n*. To deal with the situation where $S_{n}$ takes its values on a lattice, with lattice step equal to *h*, an alternative version of $m_{S_{n}}$ is constructed as $m_{S_{n} }^{cont}(s)=m_{S_{n}}((k+1)h)/h$ whenever $kh<s\le (k+1)h$. It is immediate that using $m_{S_{n}}^{cont}$ as the prior predictive to check the prior gives the same value as $M_{S_{n}}(m_{S_{n}}(s)\le m_{S_{n}}(S_{n}(x_{n})))$. It is then established (in Lemma 4 of that paper) that condition (ii) is satisfied for the binomial, with $S_{n}(x_{n})$ equal to the proportion of 1’s in the sample, and so (8) also holds. The same issue arises with the multinomial but the proof of the consistency is much more difficult, as a comparison between the arguments in Evans and Jang (2011a) and the proof of Theorem 1 in the following section demonstrates.

*Multinomial Case* As with the binomial case it is necessary to construct a continuous probability measure that is essentially equivalent to $M_{T}$. To get rid of the linear dependence among the coordinates let $T_{n}$ be the first *k* coordinates of *T* with prior predictive measure $M_{T_{n}}$, density $m_{T_{n}}$ and put ${\varTheta }_{k}^{*}=\{(\theta _{1},\ldots , \theta _{k}):\theta _{i}>0,\theta _{1}+\cdots +\theta _{k}<1\}$.

Let $D_{k,n}=\{{\mathbf {n}}=(n_{1},\ldots ,n_{k}):n_{i}\in {\mathbb {N}}_{0},n_{1}+\cdots +n_{k}\le n\}$ denote the set of possible values of $T_{n}$. We construct a set of disjoint *k*-cells that cover ${\varTheta }_{k}^{*}$ and are indexed by $\mathbf {n} \in D_{k,n}$ such that $\mathbf {n/}n$ is in the set that ${\mathbf {n}}$ indexes. Let ${\varTheta }_{k}^{*}({\mathbf {n}})= X_{i=1}^{k}[n_{i}/n-1/2n,n_{i}/n+1/2n)$ and the ${\varTheta }_{k}^{*}({\mathbf {n}})$ are disjoint, $\mathbf {n/}n$ is the center of ${\varTheta }_{k}^{*}({\mathbf {n}})$, vol$({\varTheta }_{k}^{*}({\mathbf {n}})) =n^{-k}$ and ${\varTheta }_{k}^{*} \subset \cup _{\mathbf {n} \in D_{k,n}}{\varTheta }_{k}^{*}({\mathbf {n}})\downarrow {\bar{{\varTheta }}}_{k}^{*}$ as $n\rightarrow \infty$.

For $\mathbf {r} \in \cup _{\mathbf {n} \in D_{k,n}}{\varTheta }_{k}^{*}({\mathbf {n}})$ define $m_{n}^{*}({\mathbf {r}})=n^{k}m_{T_{n}}({\mathbf {n}}({\mathbf {r}}))$ where ${\mathbf {n}}({\mathbf {r}})\in D_{k,n}$ is such that $\mathbf {r} \in {\varTheta }_{k}^{*}({\mathbf {n}}({\mathbf {r}}))$. Note that for $\mathbf {r} \in {\varTheta }_{k}^{*}({\mathbf {n}}({\mathbf {r}}))$, then ${\mathbf {n}}({\mathbf {r}} )=(n_{1}(\mathbf {r)},\ldots ,n_{k}(\mathbf {r)})$ where $n_{i}(\mathbf {r)} =\lfloor nr_{i}+1/2\rfloor$ and $\lfloor x\rfloor$ is the floor function. Also, let $c_{n}(\mathbf {r)=n}({\mathbf {r}})/n$, the center of the *k*-cell containing $\mathbf {r}$. We construct $M_{n}^{*}$ a continuized version of $M_{T_{n}}$.

### Lemma 3

(i) $||\mathbf {r}-c_{n} {({\mathbf {r}})}||\le \sqrt{k}/n$ so $c_{n}{({\mathbf {r}} )}\rightarrow {\mathbf {r}}$ as $n\rightarrow \infty$. (ii) $m_{n}^{*}$ is constant on ${\varTheta }_{k}({\mathbf {n}})$ and takes the value $n^{k}m_{T_{n} }({\mathbf {n}})$ there. (iii) $m_{n}^{*}$ is the density of an absolutely continuous probability measure $M_{n}^{*}$ where $M_{n}^{*}({\varTheta }_{k} ({\mathbf {n}}))=m_{T_{n}}({\mathbf {n}})$ and $M_{T_{n}}(\{{\mathbf {t}}:m_{T_{n} }({\mathbf {t}})\le m_{T_{n}}(T_{n}(x))\})=M_{n}^{*}(\{{\mathbf {r}}:m_{n}^{*} ({\mathbf {r}})\le m_{n}^{*}(T_{n}(x)/n)\})$.

### Proof

Parts (i) and (ii) are obvious. For (iii) we have $M_{T_{n}}(\{{\mathbf {t}}:m_{T_{n}}({\mathbf {t}})\le m_{T_{n}}(T_{n} (x))\}) =M_{T_{n}}(\{n({\mathbf {r}}):m_{n}^{*}({\mathbf {r}})\le m_{n}^{*}(T_{n}(x)/n)\})$. $\square$

For $s>0$, define $G_{n,s}({\mathbf {r}})=\{\theta \in {\varTheta }_{k}^{*}: \sum _{i=1}^{k+1}c_{ni}{({\mathbf {r}})}\log (\theta _{i}/c_{ni} {({\mathbf {r}})})>-[(k+1+s)\log (n)]/n=\{\theta \in {\varTheta }_{k}^{*}: KL(c_{n}(\mathbf {r})\,||\,\theta )<[(k+1+s)\log (n)]/n\}$, a *KL* neighborhood of $c_{n}(\mathbf {r})$. We have the following where $B_{\epsilon }({\mathbf {r}})$ is the ball of radius $\epsilon$ centered at ${\mathbf {r}}$.

### Lemma 4

If $\mathbf {r} \in {\varTheta }_{k}^{*}$, then, for $\epsilon >0$, there exists *N* such that for all $n>N,\mathbf {r}\in \, G_{n,s}({\mathbf {r}}) \subset B_{\epsilon }({\mathbf {r}})$, so $G_{n,s}({\mathbf {r}})\longrightarrow \{\mathbf {r}\}$ as $n\rightarrow \infty$.

### Proof

Note that $r_{i}\ne 0$, so for *n* large enough $$\lfloor nr_{i}+1/2\rfloor \ge 1\ $$and this holds for all *i*. Since $nr_{i}+1/2\ge \, \lfloor nr_{i}+1/2\rfloor$ and $\log (nr_{i}/(nr_{i} +1/2))<0$,

$$\begin{aligned} \sum _{i=1}^{k+1}c_{n,i}(\mathbf {r}) \log \left( \frac{r_{i}}{c_{n,i} }\right)&=\sum _{j=1}^{k+1}\left( \frac{\lfloor nr_{i}+1/2\rfloor }{n}\right) \log \left( \frac{nr_{i}}{\lfloor nr_{i}+1/2\rfloor }\right) \\&\ge \sum _{i=1}^{k+1}\left( \frac{nr_{i}+1/2}{n}\right) \log \left( \frac{nr_{i}}{nr_{i}+1/2}\right) \\&=\sum _{i=1}^{k+1}\left( \frac{nr_{i}+1/2}{n}\right) \log \left( 1-\frac{1}{2(nr_{i}+1/2)}\right) \\&=-\frac{1}{2n}\sum _{i=1}^{k+1}\sum _{j=1}^{\infty }\frac{\left[ 2(nr_{i}+1/2)\right] ^{-j+1}}{j} \\&\ge -\frac{1}{2n}\sum _{i=1}^{k+1} \sum _{j=1}^{\infty }\left[ 2(nr_{i}+1/2)\right] ^{-j+1}\\&=-\frac{1}{2n}\sum _{i=1}^{k+1}\frac{\left[ 2(nr_{i}+1/2)\right] ^{-2} }{1-1/2(nr_{i}+1/2)} \\&\ge -\frac{1}{2n}\sum _{i=1}^{k+1}\left( 1+\frac{1}{2nr_{i}}\right) \\&=-\frac{k+1}{2n}-\frac{1}{4n^{2}}\sum _{i=1}^{k+1}\frac{1}{r_{i}} \end{aligned}$$

and clearly there is an *N* such that this is bounded below by $-[(k+1+s)\log (n)]/n$ for all $n>N$. Therefore, $\mathbf {r} \in G_{n,s}({\mathbf {r}})$ for all *n* large enough. If $||\mathbf {r}-\theta ||=\epsilon >0$, then by Lemma 3 (i), $||c_{n}{({\mathbf {r}})-}\theta ||>\epsilon /2$ for all *n* large enough. As $\sum _{j=1}^{k+1}c_{nj}{({\mathbf {r}})}\log (\theta _{j}/c_{nj} {({\mathbf {r}})})$ is continuous in $c_{n}{({\mathbf {r}})}$, bounded above by 0 and 0 iff $c_{n}{({\mathbf {r}})=} \,\, \theta$, then we have that $\sum _{j=1}^{k+1}c_{nj}{({\mathbf {r}})}\log (\theta _{j} /c_{nj}{({\mathbf {r}})})\rightarrow \delta <0$. Conclude that $\theta {\notin }G_{n,s}({\mathbf {r}})$ for all *n* large enough. $\square$

If ${\mathbf {r}}{\notin }{\bar{{\varTheta }}}_{k}^{*}$, then $m_{n}^{*} ({\mathbf {r}})\rightarrow \pi ({\mathbf {r}})=0$. We prove that $m_{n}^{*} ({\mathbf {r}})\rightarrow \pi ({\mathbf {r}})$ when $\mathbf {r} \in {\varTheta }_{k,c}^{*}$.

### Lemma 5

If $\pi$ satisfies A1, A2 then, for $\mathbf {r} \in {\varTheta }_{k}^{*}$, any $s>0$ and $n>(1+Bk!)^{2} ,|m_{n}^{*}({\mathbf {r}})-n!n^{k}\pi ({\mathbf {r}})/(n+k)!|\le n^{-s} +\sup \nolimits _{{\theta }\in G_{n,s}({\mathbf {r}})\cap {\varTheta }_{k,c}^{*}} |\pi (\theta )-\pi ({\mathbf {r}})|$.

### Proof

Put $$G_{n,s}=G_{n,s}({\mathbf {r}}),F_{n,s}={\varTheta }_{k}-G_{n,s}, w_{{\mathbf {n}}}(\theta )=n!\prod _{j>0}\theta _{j}^{n_{j}}/n_{j}!$$, then

$$\begin{aligned} \left| m_{n}^{*}({\mathbf {r}})-\frac{n!n^{k}}{(n+k)!}\pi ({\mathbf {r}})\right|&=\left| n^{k}\int _{{\varTheta }_{k}}w_{{\mathbf {n}} }(\theta )\,{\varPi }(d\theta )-n^{k}\pi ({\mathbf {r}})\int _{{\varTheta }_{k}}w_{{\mathbf {n}} }(\theta )\,d\theta \right| \\&\le n^{k}\int _{F_{n,s}}w_{{\mathbf {n}}}(\theta )\,{\varPi }(d\theta ) +\pi ({\mathbf {r}})n^{k}\int _{F_{n,s}}w_{{\mathbf {n}}}(\theta )\,{\varPi }(d\theta )\\&\quad +n^{k}\int _{G_{n,s}} w_{{\mathbf {n}}}(\theta )|\pi (\theta )-\pi ({\mathbf {r}})|\,d\theta \end{aligned}$$

and we now bound the integrals $I_{n,1},I_{n,2},I_{n,3}$ in this expression. For $\theta \in F_{n,s}$, since $n_{j}=nc_{n,j},\sum _{j=1}^{k+1}n_{j} \log (\theta _{j})\le \sum _{j=1}^{k+1}n_{j}\log (n_{j})-(n+k+1+s)\log (n)$,

$$\begin{aligned} n!\prod _{j=1}^{k+1}\frac{1}{n_{j}!}\times \prod _{j=1}^{k+1}\theta _{j}^{n_{j} }\le & {} n!\prod _{j=1}^{k+1}\frac{1}{n_{j}!}\times \frac{1}{n^{n}} \prod _{j=1}^{k+1}n_{j}^{n_{j}}\times \frac{1}{n^{k+1+s}} \\= & {} \frac{n!}{n^{n}} \prod _{n_{j}>0}\frac{n_{j}^{n_{j}}}{n_{j}!}\times \frac{1}{n^{k+1+s}}. \end{aligned}$$

When $\max (n_{1},\ldots ,n_{k+1})=n$, then $(n!/n^{n})\prod _{j:n_{j}>0} (n_{j}^{n_{j}}/n_{j}!)=1\le n^{1/2}$. Now assume $\max (n_{1},\ldots ,n_{k+1})<n$. Since $1/(12n+1)<\log (n!)-\frac{1}{2}\log (2\pi n)+n\log (n)-n<1/12n$ for all $n>0$ by Robbins (1955),

$$\begin{aligned} n^{-n}w_{{\mathbf {n}}}(\theta )\le (2\pi n)^{1/2}e^{n}e^{1/12n}\prod _{n_{j}>0}\frac{e^{-n_{j}}e^{-1/(12n_{j}+1)}}{(2\pi n_{j})^{1/2}}\le \frac{n^{1/2} }{(2\pi )^{1/2}}\prod _{n_{j}>0}\frac{1}{n_{j}^{1/2}}<n^{1/2} \end{aligned}$$

since $0<\exp \{1/12n-\sum _{j:n_{j}>0}1/(12n_{j}+1)\}<1$. So for any prior ${\varPi }$, we have that $n^{k}\int _{F_{n,s}}w_{{\mathbf {n}}}(\theta )\,{\varPi }(d\theta )\le n^{k}\int _{F_{n,s}}n^{1/2}n^{-(k+1+s)}\,{\varPi }(d\theta )\le n^{-(s+1/2)}$ hence $I_{n,1}\le n^{-s-1/2}$ regardless of prior. The Dirichlet$(1,\ldots ,1)$ has density *k*! on ${\varTheta }$. The above argument with this prior implies $I_{n,2}\le \pi ({\mathbf {r}})k!n^{-s-1/2}$. By A2, $I_{n,3}=n^{k}\int _{G_{n,s}\cap {\varTheta }_{k,c}}w_{{\mathbf {n}}}(\theta )|\pi (\theta )-\pi ({\mathbf {r}})|\,d\theta$ which, using $\int _{{\varTheta }_{k} }w_{{\mathbf {n}}}(\theta )\,d\theta =n!/(n+k)!$, implies

$$\begin{aligned} I_{n,3}\le (n!n^{k}/(n+k)!)\sup \nolimits _{{\theta }\in G_{n,s}\cap {\varTheta }_{k,c}}|\pi (\theta )-\pi ({\mathbf {r}})|\le \sup \nolimits _{{\theta }\in G_{n,s}\cap {\varTheta }_{k,c}}|\pi (\theta )-\pi ({\mathbf {r}})| \end{aligned}$$

since $n!n^{k}/(n+k)!\le 1$. Finally, for $n\ge (1+Bk!)^{2}$ and using A1, $I_{n,1}+I_{n,2}+I_{n,3}$ has the following upper bound establishing the result,

$$\begin{aligned} \frac{1+Bk!}{n^{1/2}}\frac{1}{n^{s}}+\sup _{{\theta }\in G_{n,s} \cap {\varTheta }_{k,c}}|\pi (\theta )-\pi ({\mathbf {r}})|\le \frac{1}{n^{s}} +\sup _{{\theta }\in G_{n,s}\cap {\varTheta }_{k,c}}|\pi (\theta )-\pi ({\mathbf {r}})|. \end{aligned}$$

$$\square $$

### Corollary 2

As $n\rightarrow \infty$, then $m_{n}^{*}\overset{a.s.}{\rightarrow }\pi$ with respect to volume measure.

### Proof

If $\mathbf {r}\notin {\bar{{\varTheta }}}_{k}$, then $m_{n}^{*}({\mathbf {r}})\rightarrow \pi ({\mathbf {r}})=0$. If $\mathbf {r}\in {\varTheta }_{k,c}^{*}$ then for $\epsilon >0 \, \exists \, \delta$ such that $\theta {\in }B_{\delta }({\mathbf {r}})$ implies $|\pi (\theta )-\pi ({\mathbf {r}})|<\epsilon /3$. By Lemma 4 there exists $N>(1+Bk!)^{2}$ such that for all $n>N$, then $G_{n,s}(\mathbf {r})\subset B_{\delta }({\mathbf {r}} ),n^{-s}<\epsilon /3$ and $1-n!n^{k}/(n+k)!<\epsilon /3B$. By Lemma 5, $|m_{n}^{*}({\mathbf {r}})-\pi ({\mathbf {r}})|\le |m_{n}^{*}({\mathbf {r}} )-n!n^{k}\pi ({\mathbf {r}})/(n+k)!|+|n!n^{k}/(n+k)!-1)|\pi ({\mathbf {r}})<\epsilon$ establishing convergence everywhere except on ${\varTheta }_{k,d}\cup \partial {\varTheta }_{k}$ which has volume 0. $\square$

The following is required for the proof of the theorem.

### Lemma 6

If $\theta ^{\text {true}}\in {\varTheta }_{k,c}$, then $\sup _{{\theta }\in G_{n,s}(T_{n}(x)/n)\cap {\varTheta }_{k,c}}|\pi (\theta )-\pi (\theta ^{\text {true}})|\rightarrow 0$ as $n\rightarrow \infty$.

### Proof

Since $\theta ^{\text {true}}\in {\varTheta }_{k,c}$, $\exists \, \delta >0$ so that, if $\theta \in B_{\delta }(\theta ^{\text {true}})$, then $|\pi (\theta )-\pi (\theta ^{\text {true}})|<\epsilon$. If $\theta \in G_{n,s}(T_{n}(x)/n)$, the Kullback–Csiszar–Kemperman inequality (Devroye 1987) says $\sum _{j=1}^{k+1}|\theta _{j}-T_{n,j}(x)/n|\le \left[ 2KL(T_{n}(x)/n{\,||\,}\theta )\right] ^{1/2}<\left[ 2n^{-1}(k+1+s)\log n\right] ^{1/2}$ implying $\sum _{j=1}^{k+1}|\theta _{j}- \theta _{j}^{\text {true}}|\le (2n^{-1}(k+1+s)\log n)^{1/2}+\sum _{j=1}^{k+1}| \theta _{j}^{\text {true}}-T_{n,j}(x)/n|$. Therefore, $T_{n}(x)/n\overset{a.s.}{\rightarrow }\theta ^{\text {true}}$ implies $\exists \, N$ such that for all $n>N$, if $\theta \in G_{n,s}(T_{n}(x)/n)$, then $||\theta - \theta ^{\text {true}}||<\delta$ proving the lemma. $\square$

The main result is now established.

### Proof of Theorem 1

Fix $0<\eta <1$ small. Under A3, $\pi (\theta )$ has a continuous distribution when $\theta {\sim }\pi$. Therefore, $\exists \, \epsilon >0$ so that ${\varPi }(\{\theta :|\pi (\theta )-\pi (\theta ^{\text {true}})|\le \epsilon \})<\eta$.

Define $H_{n}=\{{\mathbf {r}}\in {\varTheta }_{k,c}:\sup _{{\theta }\in G_{n,s}({\mathbf {r}})\cap {\varTheta }_{k,c}}|\pi (\theta )-\pi ({\mathbf {r}})|<\epsilon /6\}$. By Lemma 4 the diameter of $G_{n,s}({\mathbf {r}})$ shrinks to zero. If ${\mathbf {r}}\in {\varTheta }_{k,c}$, then $\sup _{{\theta }\in G_{n,s} ({\mathbf {r}})\cap {\varTheta }_{k,c}}|\pi (\theta )-\pi ({\mathbf {r}})|\rightarrow 0$ as $n\rightarrow \infty$, so there exists $N({\mathbf {r}},\epsilon )>0$ such that ${\mathbf {r}}\in H_{n}$ for all $n\ge N({\mathbf {r}},\epsilon )$. This implies $H_{n}\uparrow {\varTheta }_{k,c}$ and ${\varPi }(H_{n})\uparrow {\varPi }({\varTheta }_{k,c})=1$ as $n\rightarrow \infty$.

From the continuity of $\pi$ at $\theta ^{\text {true}}$, there exists $\delta >0$ such that $|\pi (\theta )-\pi (\theta ^{\text {true}})|<\epsilon /12$ for any $\theta \in B_{\delta }(\theta ^{\text {true}})$. The strong law of large numbers implies $T_{n}(x)/n\rightarrow \theta ^{\text {true}}$ almost surely so there exits $N_{1}$ such that $||T_{n}(x)/n-\theta ^{\text {true}}||<\delta$ for all $n>N_{1}$. Therefore, if $n>N_{1}$, then $|\pi (T_{n}(x)/n)-\pi (\theta ^{\text {true}})|<\epsilon /12$. Also, by Lemma 6 there exists $N_{2}$ such that for $n>N_{2}$, then $\sup _{{\theta }\in G_{n,s}(T_{n} (x)/n)\cap {\varTheta }_{k,c}}\left| \pi (\theta )-\pi (\theta ^{\text {true} })\right| <\epsilon /6$ and $n^{-s}<\epsilon /6$. Therefore, using Lemma 5, for all $n>N_{3}=\max \{(1+B_{m}k!)^{2},N_{1},N_{2}\}$,

$$\begin{aligned}&|m_{n}^{*}\left( T_{n}(x)/n\right) -n!n^{k}\pi (\theta ^{\text {true} })/(n+k)!|\\&\quad \le \left| m_{n}^{*}\left( \frac{T_{n}(x)}{n}\right) -\frac{n!n^{k}}{(n+k)!}\pi \left( \frac{T_{n}(x)}{n}\right) \right| +\frac{n!n^{k}}{(n+k)!}\left| \pi \left( \frac{T_{n}(x)}{n}\right) -\pi (\theta ^{\text {true}})\right| \\&\quad \le n^{-s}+\sup \nolimits _{{\theta }\in G_{n,s}(T_{n}(x)/n)\cap {\varTheta }_{k,c}}\left| \pi (\theta )-\pi \left( T_{n}(x)/n\right) \right| +\epsilon /12\\&\quad \le \epsilon /6+\sup \nolimits _{{\theta }\in G_{n,s}(T_{n} (x)/n)\cap {\varTheta }_{k,c}}|\pi (\theta )-\pi (\theta ^{\text {true}})||\le \epsilon /2. \end{aligned}$$

Note that this implies that for all $n>N_{3}$,

9 $$\begin{aligned} m_{n}^{*}(T_{n}(x)/n)\le n!n^{k}\pi (\theta ^{\text {true}})/(n+k)!+\epsilon /2\le \pi (\theta ^{\text {true}})+\epsilon /2. \end{aligned}$$

Now $M_{n}^{*}(\{{\mathbf {r}}:m_{n}^{*}({\mathbf {r}})\le m_{n}^{*} (T_{n}(x)/n)\})=M_{n}^{*}(\{{\mathbf {r}}:m_{n}^{*}({\mathbf {r}})\le m_{n}^{*}(T_{n}(x)/n)\}\cap {\varTheta }_{k,c})$ since $M_{n}^{*}$ is absolutely continuous and ${\varTheta }_{k,d}$ has volume measure 0. Also,

$$\begin{aligned} M_{n}^{*}(\{{\mathbf {r}}:m_{n}^{*}({\mathbf {r}})\le m_{n}^{*} (T_{n}(x)/n)\}\cap {\varTheta }_{k,c})&= M_{n}^{*}(\{{\mathbf {r}}:m_{n}^{*}({\mathbf {r}})\le m_{n}^{*}(T_{n}(x)/n)\}\cap H_{n})\\&\quad +M_{n}^{*}(\{{\mathbf {r}} :m_{n}^{*}({\mathbf {r}})\le m_{n}^{*} (T_{n}(x)/n)\}\cap H_{n}^{c}\cap {\varTheta }_{k,c}) \end{aligned}$$

and $M_{n}^{*}(\{{\mathbf {r}}:m_{n}^{*}({\mathbf {r}})\le m_{n}^{*} (T_{n}(x)/n)\}\cap H_{n}^{c}\cap {\varTheta }_{k,c})\le M_{n}^{*}(H_{n}^{c} \cap {\varTheta }_{k,c})$. Using Lemma 5 and the bound $|\pi ({\theta } )-\pi ({\mathbf {r}})|\le 2B$, then when $n>(1+Bk!)^{2}$

$$\begin{aligned} M_{n}^{*}(H_{n}^{c}\cap {\varTheta }_{k,c})\le (n!n^{k}/(n+k)!){\varPi }(H_{n}^{c} \cap {\varTheta }_{k,c})+n^{-s}+2Bvol(H_{n}^{c}\cap {\varTheta }_{k,c}). \end{aligned}$$

So $M_{n}^{*}(H_{n}^{c}\cap {\varTheta }_{k,c})\rightarrow 0$ and $M_{n}^{*}(\{{\mathbf {r}}:m_{n}^{*}({\mathbf {r}})\le m_{n}^{*}(T_{n}(x)/n)\}\cap H_{n}^{c}\cap {\varTheta }_{k,c})\rightarrow 0$ as $n\rightarrow \infty$.

Now put $A_{n}=M_{n}^{*}(\{{\mathbf {r}}\in H_{n}:m_{n}^{*}({\mathbf {r}})\le m_{n}^{*}(T_{n}(x)/n)\})$. There exist $N_{4}>N_{3}$ such that for all $n>N_{4}$, then $0<(1-n!n^{k}/(n+k)!)B\le \epsilon /6$. Then, for all $n>N_{4}$, by Lemma 5 and the definition of $H_{n}$, for all ${\mathbf {r}}\in H_{n}$,

10 $$\begin{aligned} m_{n}^{*}({\mathbf {r}})&\ge \frac{n!n^{k}}{(n+k)!}\pi ({\mathbf {r}} )-\frac{1}{n^{s}}-\sup _{{\theta }\in G_{n,s}({\mathbf {r}}) \cap {\varTheta }_{k,c}}|\pi (\theta )-\pi ({\mathbf {r}})| \nonumber \\&= \pi ({\mathbf {r}})- \left( 1-\frac{n!n^{k}}{(n+k)!}\right) \pi ({\mathbf {r}})-\frac{1}{n^{s}} \nonumber \\&\quad -\sup _{{\theta }\in G_{n,s}({\mathbf {r}} )\cap {\varTheta }_{k,c}}|\pi (\theta )-\pi ({\mathbf {r}})|\ge \pi ({\mathbf {r}})-\epsilon /2. \end{aligned}$$

By (9) and (10), for all $n>N_{4}$,

$$\begin{aligned} A_{n}\le M_{n}^{*}(\{{\mathbf {r}}\in H_{n}:\pi ({\mathbf {r}})\le \pi (\theta ^{\text {true}})+\epsilon \})\le M_{n}^{*}(\{{\mathbf {r}} :\pi ({\mathbf {r}})\le \pi (\theta ^{\text {true}})+\epsilon \}). \end{aligned}$$

Putting $C_{\epsilon }(\theta ^{\text {true}})=\{{\mathbf {r}}:\pi ({\mathbf {r}} )\le \pi (\theta ^{\text {true}})+\epsilon \}$, we have, as $n\rightarrow \infty$

$$\begin{aligned} \left| M_{n}^{*}(C_{\epsilon }(\theta ^{\text {true}}))-{\varPi }(C_{\epsilon }(\theta ^{\text {true}})\right|&=\left| \int _{C_{\epsilon }(\theta ^{\text {true}})}\left( m_{n}^{*}(\theta )-\pi (\theta )\right) \,d\theta \right| \\&\le \int _{{\varTheta }_{k}}\left| m_{n}^{*}(\theta )-\pi (\theta )\right| \,d\theta \rightarrow 0 \end{aligned}$$

by Corollary 2 and Scheffé’s theorem and so, $\limsup _{{}}A_{n}\le {\varPi }(\pi ({\mathbf {r}})\le \pi (\theta ^{\text {true}})+\epsilon )$. Similarly, $\liminf A_{n}\ge {\varPi }(\pi ({\mathbf {r}})\le \pi (\theta ^{\text {true}})-\epsilon )$. Therefore, $\limsup A_{n}-\liminf A_{n}\le {\varPi }(|\pi (\theta )-\pi (\theta ^{\text {true}})|\le \epsilon )\le \eta$. Since $\eta >0$ is arbitrary, $\lim _{n\rightarrow \infty }A_{n} \, ={\varPi }(\{{\mathbf {r}}\in {\varTheta }_{k,c} :\pi ({\mathbf {r}})\le \pi (\theta ^{\text {true}})\})$ and the proof is complete. $\square$
